# Supplementary material for: Unsupervised discovery of behaviorally relevant brain states in rats playing hide-and-seek
Source: Curr Biol. 2022 Jun 20;32(12):2640–2653.e4. doi: 10.1016/j.cub.2022.04.068 (PMC9245901; doi:10.1016/j.cub.2022.04.068)
Supplement: Document S1. Figures S1–S6 [file mmc1.pdf]

Current Biology, Volume *32*

Supplemental Information

Unsupervised discovery of behaviorally relevant brain states in rats playing hide-and-seek

Bence Bagi, Michael Brecht, and Juan Ignacio Sanguinetti-Scheck

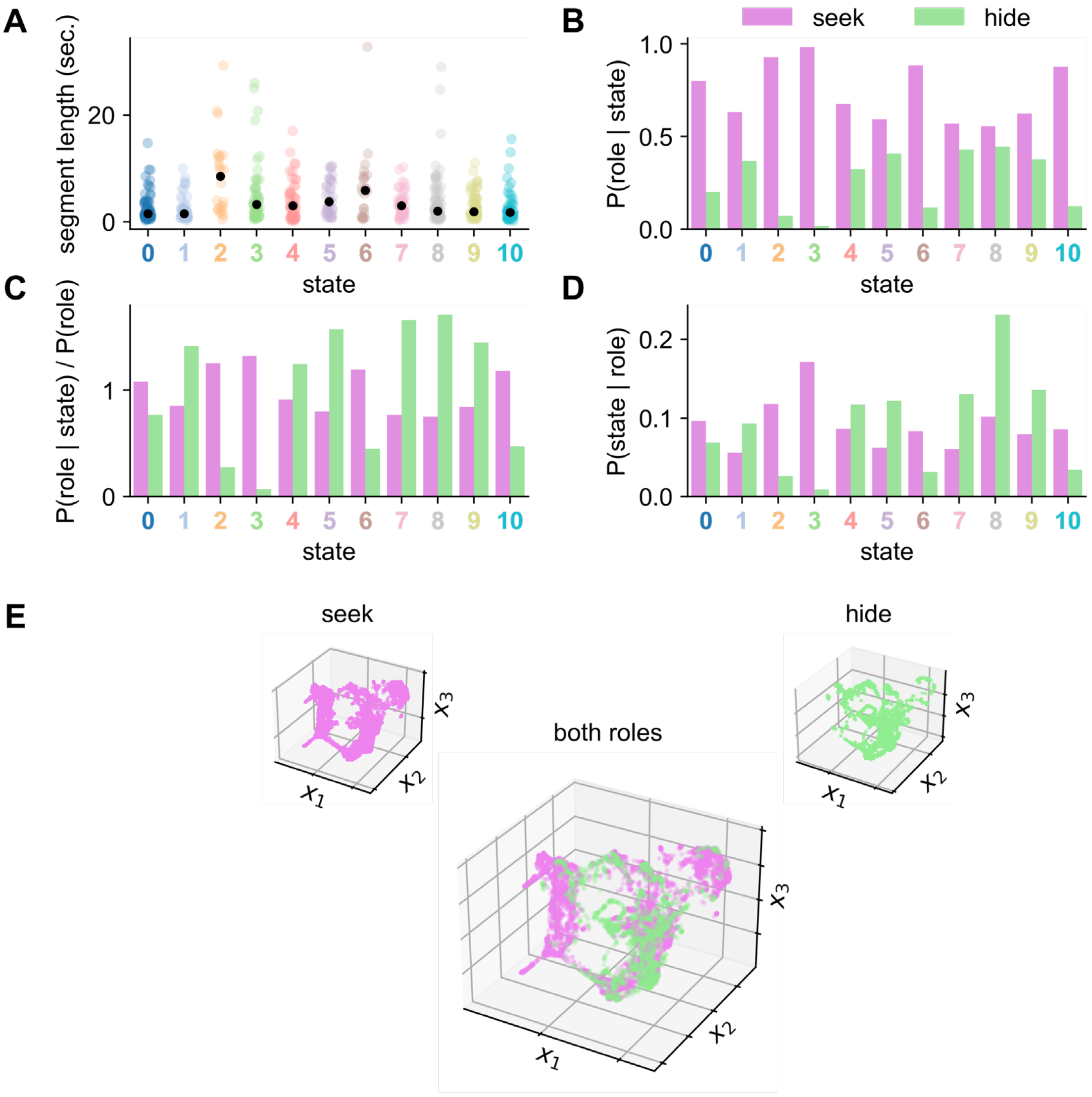

Figure S1) State segment lengths and state usage in the hide-and-seek roles in the reference session. **Related to Figure 2. A)** Segment lengths for each HMM state. Median values indicated in black. **B)** P(role | state) **C)** P(role | state) / P(role). This normalization takes into account that seek trials are longer and hence more probable. **D)** P(state | role) **E)** UMAP embeddings of neural activity colored by hide-and-seek role.

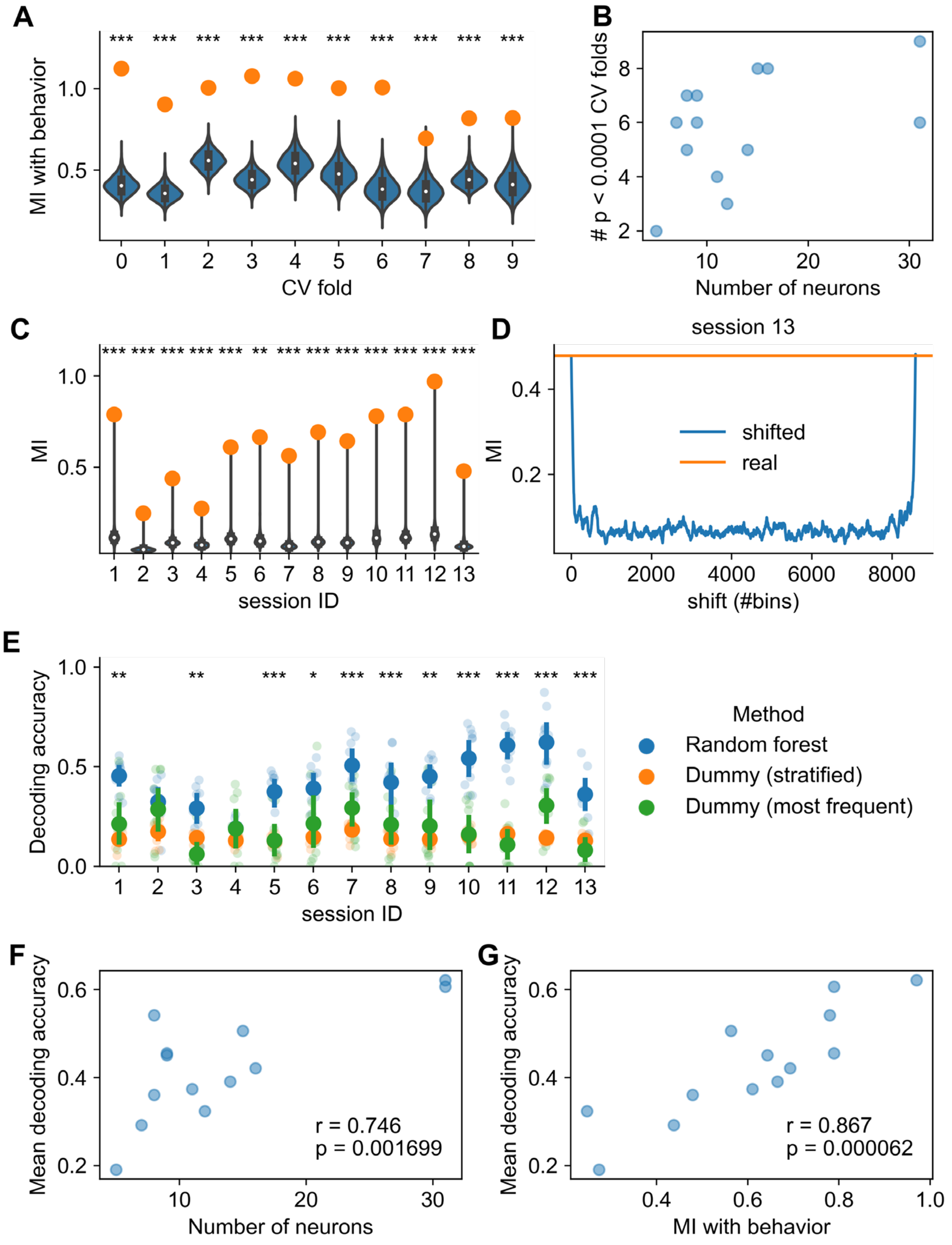

Figure S2) Cross-validated mutual information analysis and supervised decoding of behavioral states. **Related to Figure 2. A)** Fitting an HMM on a subset of trials, then applying on held-out

trials and performing the same shuffling analysis as in Fig 2E on the reference session. The star-code shows the p-value for the bootstrap with *=0.05, **=0.01, ***=0.001 **B)** Number of significant cross-validation folds (out of 10 folds) per session as a function of the number of recorded neurons in that session (using $p < 0.0001$, as this level was found to be true for every session without cross-validation in Fig 2E). **C)** Mutual information of HMM states vs behavior analysis (orange dot) for all sessions compared to a circular shuffling of the HMM states by all possible time-shifts (blue distribution) **D)** Figure shows for session #12 how the consecutive shifts affect mutual information. **E)** Cross-validated decoding of behavioral states from GPFA factors (10-fold CV across trials) using a random-forest classifier performs better than chance (performance of dummy classifiers) (one-sided Wilcoxon signed-rank test). The star-code shows the p-value with *=0.05, **=0.01, ***=0.001 **F)** Mean decoding accuracy increases with the number of neurons. (Pearson correlation, one-sided test). **G)** Mean decoding accuracy increases with the MI with behavior on the same session. (Pearson correlation, one-sided test.)

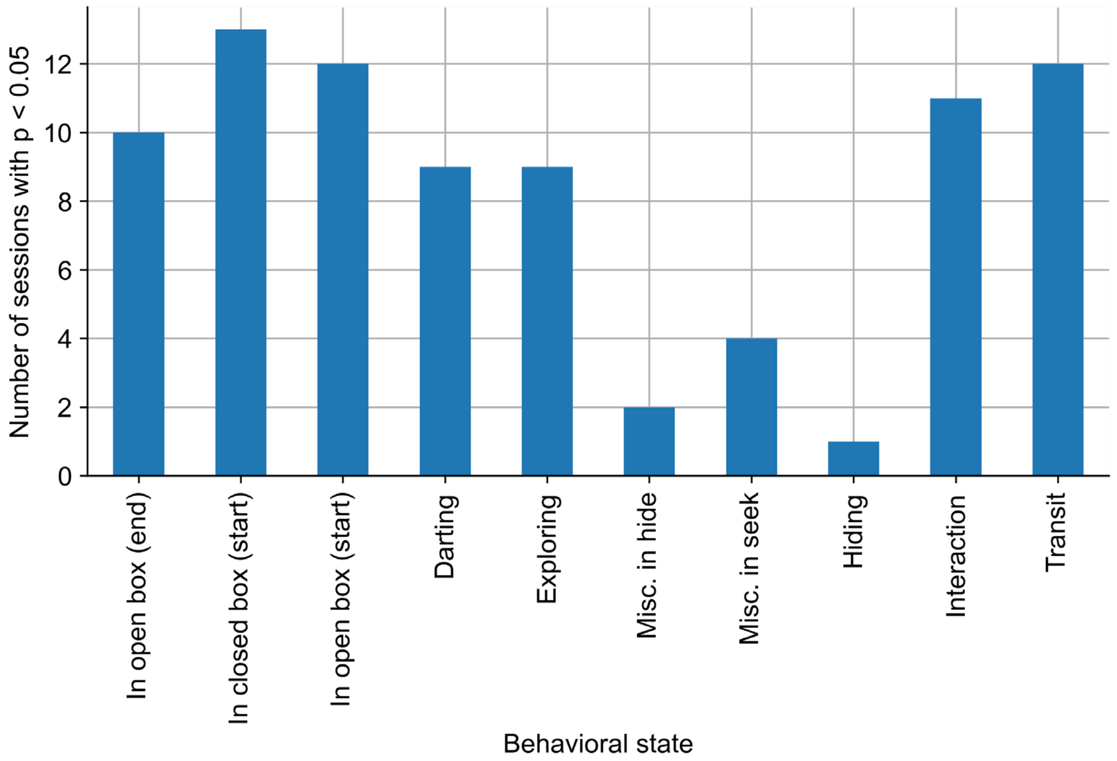

Figure S3) Significant sessions per behavior. Related to Figure 3. The number of sessions (out of 13) in which there is an HMM state significantly correlated with a tagged behavioral state ($p < 0.05$, Holm-Bonferroni correction for multiple comparisons)

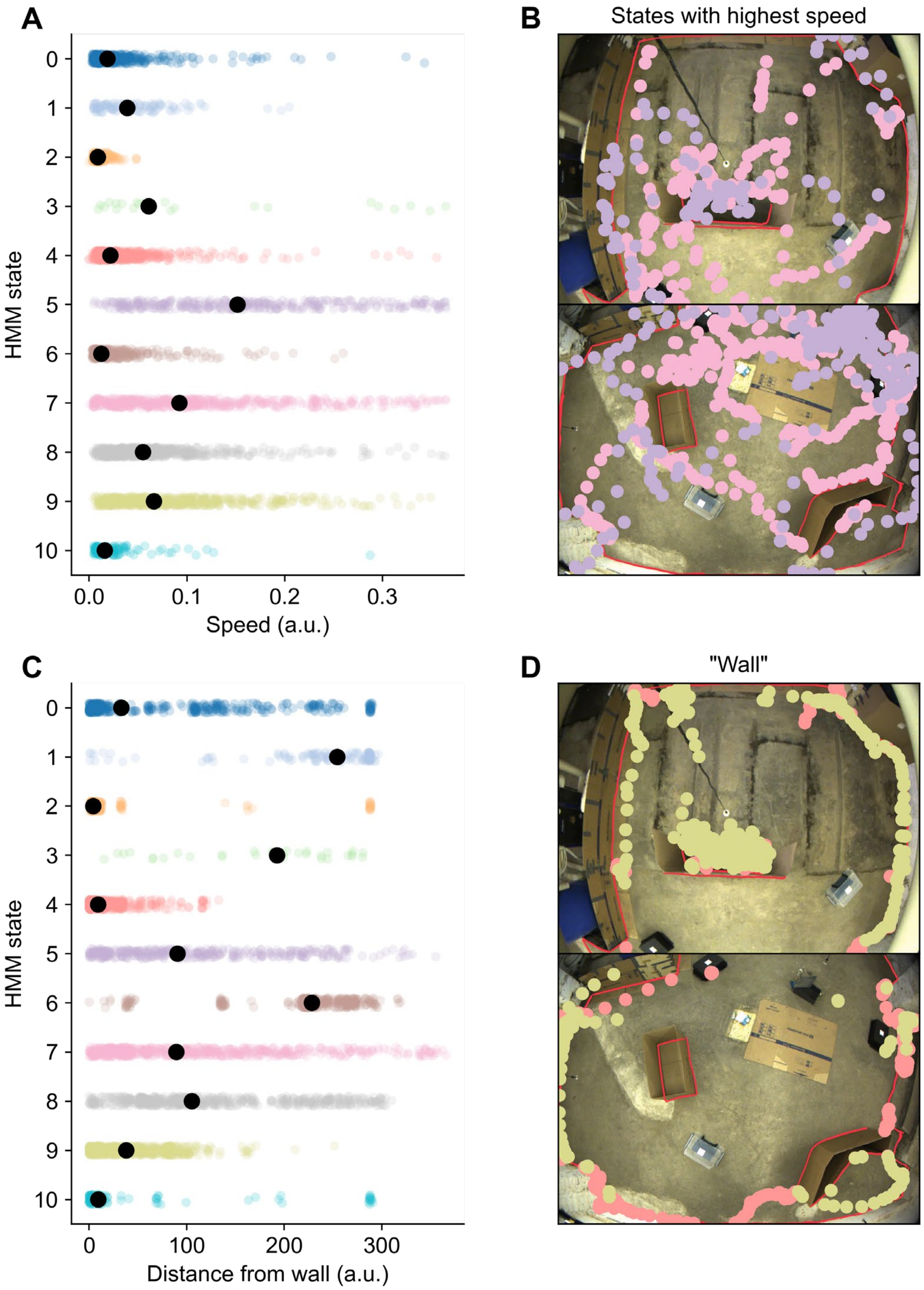
A
B
States with highest speed
HMM state
0
1
2
3
4
5
6
7
8
9
10
0.0
0.1
0.2
0.3
Speed (a.u.)
C
D
"Wall"
HMM state
0
1
2
3
4
5
6
7
8
9
10
0
100
200
300
Distance from wall (a.u.)

Figure S4) Quantification of the "wall" and "running" states. Related to Figure 4. A) Animal's approximate speed when each state was active. One data point per timestep. **B)** Animal's position when states #7 (running) and #5 (transit) were active. **C)** Animal's approximate distance from the nearest wall when each state was active. **D)** Animal's positions when states #9 and #4 were active. These states were active when the animal was walking along (#9 and #4) and stopping at (#4) walls.

A

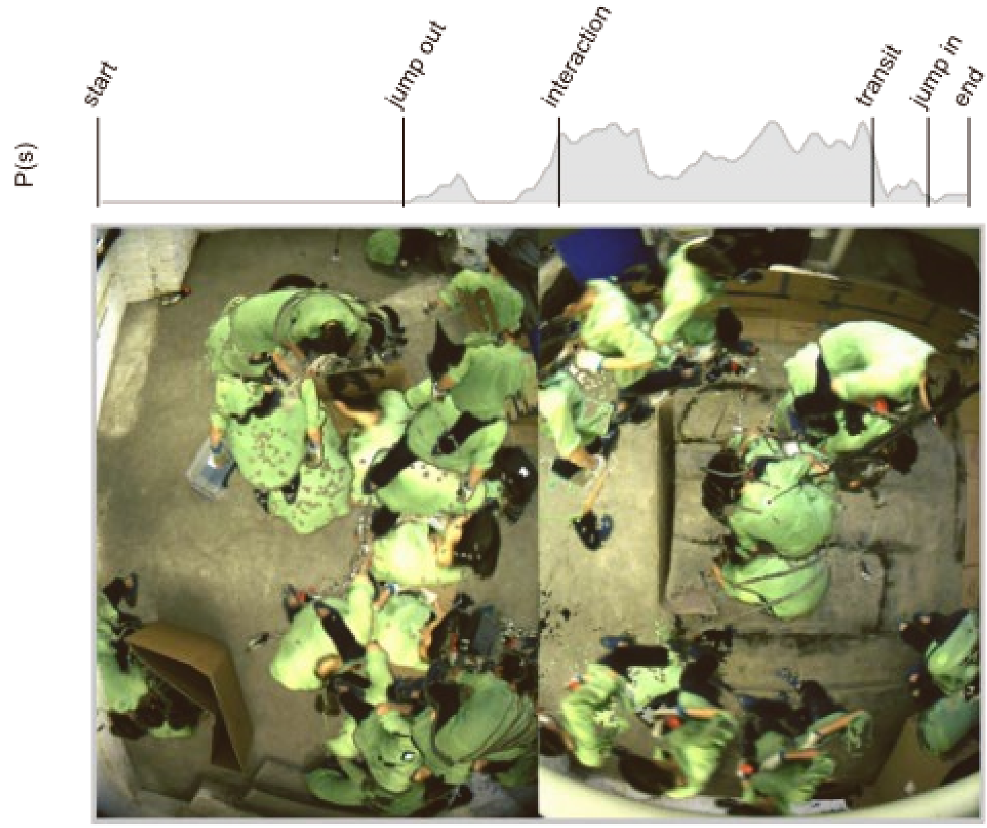
start
jump out
interaction
transit
jump in
end
P(s)

B

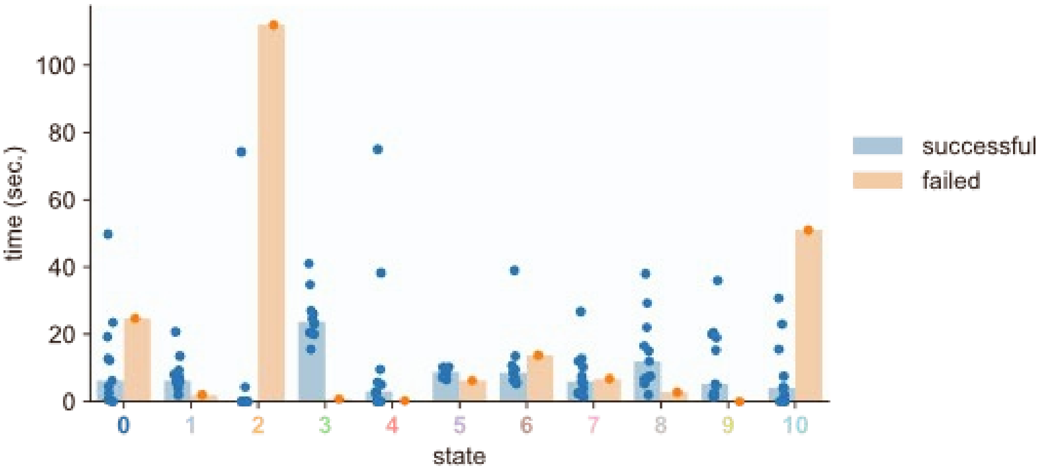
time (sec.)
100
80
60
40
20
0
0
1
2
3
4
5
6
7
8
9
10
state
successful
failed

Figure S5) Interaction-related state and its absence in failed trials. Related to Figure 4. A) Condensed video frames during state #8 show interaction with the experimenter. Marginal: probability of the state #8 throughout the trial, averaged across all trials of the reference session. **B)** Time spent in the different states for the reference session on failed and successful trials of seek behavior. Note that for successful trials state #8 (playing with the experimenter) has significantly longer durations than for the single failed seek trial. Note that the failed trial is dominated by states #2 and #10 that we have post-hoc discovered as resting states.

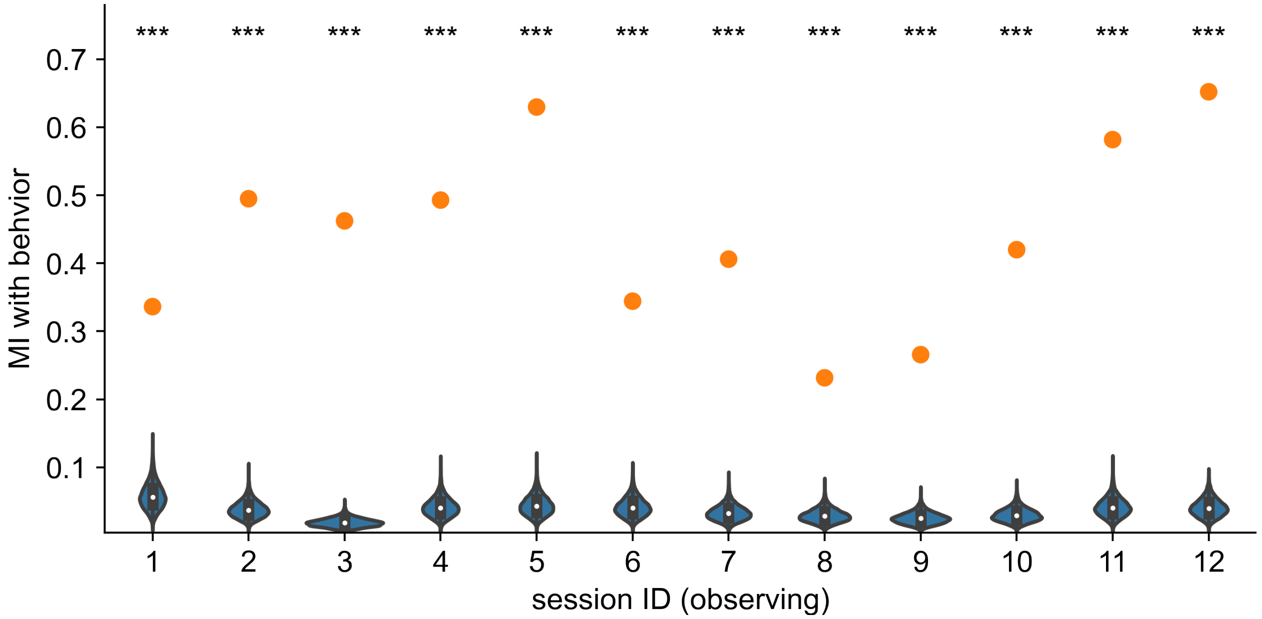

Figure S6) Separation between playing and observing. Related to Figure 6. The separation between playing- and observing states, quantified by mutual information, is well above chance level ($p < 0.0001$ in all sessions). The star-code shows the p-value for the bootstrap with *=0.05, **=0.01, ***=0.001
