# Supplementary material for: Unsupervised discovery of behaviorally relevant brain states in rats playing hide-and-seek
Source: Curr Biol. 2022 Jun 20;32(12):2640–2653.e4. doi: 10.1016/j.cub.2022.04.068 (PMC9245901; doi:10.1016/j.cub.2022.04.068)
Supplement: Document S2. Article plus supplemental information [file mmc8.pdf]

Article

Current Biology

Unsupervised discovery of behaviorally relevant brain states in rats playing hide-and-seek

Graphical abstract

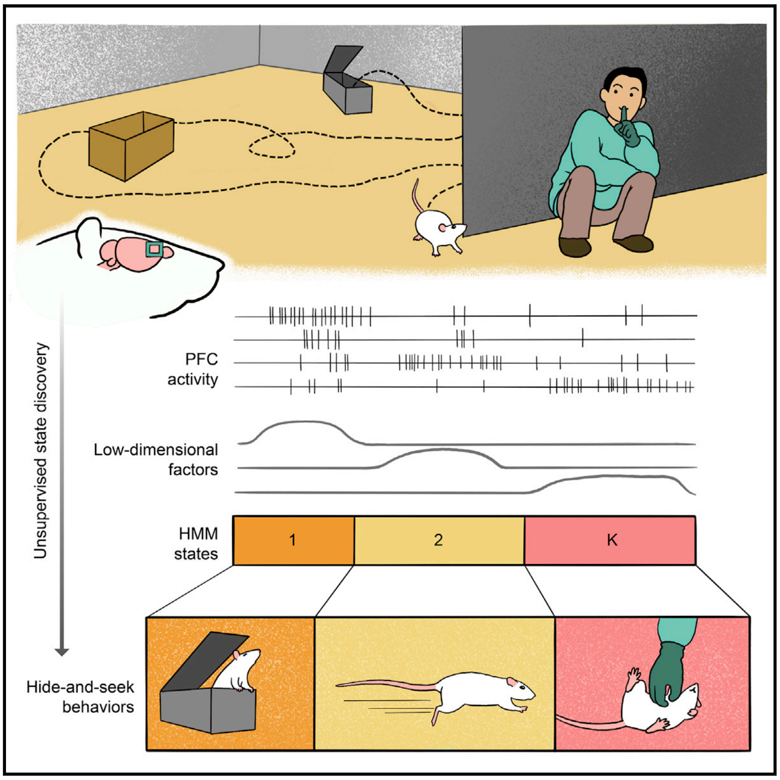

Highlights

- We used hidden Markov models to cluster neural activity of rats playing hide-and-seek
- Inferred latent states follow the phases of the hide-and-seek game
- Reverse physiology of latent states allows discovery of neurally relevant behaviors
- Rat prefrontal cortex is in distinct sets of states while playing and observing play

Authors

Bence Bagi, Michael Brecht,
Juan Ignacio Sanguinetti-Scheck

Correspondence

jsanguinettischeck@fas.harvard.edu

In brief

Bagi et al. use hidden Markov models to study PFC activity of rats playing hide-and-seek with humans. Such models, while naive and unsupervised, segment neural activity into meaningful states. States found in PFC activity alone correlate with hide-and-seek behaviors and allow for the discovery of previously undetected behaviors.

Bagi et al., 2022, Current Biology *32*, 2640–2653
June 20, 2022 © 2022 The Authors. Published by Elsevier Inc.
https://doi.org/10.1016/j.cub.2022.04.068

CellPress

CellPress
OPEN ACCESS

Current Biology

Article

Unsupervised discovery of behaviorally relevant brain states in rats playing hide-and-seek

Bence Bagi,[1,2] Michael Brecht,[1,3] and Juan Ignacio Sanguinetti-Scheck[1,4,5,6,*]
[1]Bernstein Center for Computational Neuroscience, Humboldt-Universität zu Berlin, Philippstr. 13, Haus 6, 10115 Berlin, Germany
[2]Department of Bioengineering, Imperial College London, London, UK
[3]NeuroCure Cluster of Excellence, Humboldt-Universität zu Berlin, Berlin, Germany
[4]Department of Organismic and Evolutionary Biology, Harvard University, Cambridge, MA 02138, USA
[5]Twitter: @neuroetho
[6]Lead contact
*Correspondence: jsanguinettischeck@fas.harvard.edu
https://doi.org/10.1016/j.cub.2022.04.068

SUMMARY

In classical neuroscience experiments, neural activity is measured across many identical trials of animals performing simple tasks and is then analyzed, associating neural responses to pre-defined experimental parameters. This type of analysis is not suitable for patterns of behavior that unfold freely, such as play behavior. Here, we attempt an alternative approach for exploratory data analysis on a single-trial level, applicable in more complex and naturalistic behavioral settings in which no two trials are identical. We analyze neural population activity in the prefrontal cortex (PFC) of rats playing hide-and-seek and show that it is possible to discover what aspects of the task are reflected in the recorded activity with a limited number of simultaneously recorded cells ($\leq$ 31). Using hidden Markov models, we cluster population activity in the PFC into a set of neural states, each associated with a pattern of neural activity. Despite high variability in behavior, relating the inferred states to the events of the hide-and-seek game reveals neural states that consistently appear at the same phases of the game. Furthermore, we show that by applying the segmentation inferred from neural data to the animals' behavior, we can explore and discover novel correlations between neural activity and behavior. Finally, we replicate the results in a second dataset and show that population activity in the PFC displays distinct sets of states during playing hide-and-seek and observing others play the game. Overall, our results reveal robust, state-like representations in the rat PFC during unrestrained playful behavior and showcase the applicability of population analyses in naturalistic neuroscience.

INTRODUCTION

Neuroscience is traditionally conducted under tight experimental control, requiring animals to engage in simple overtrained behaviors. Natural behavior, however, is more complex. Although early approaches to the study of natural behavior based on observation separated animal behavior into different states defining an ethogram,[1] neuroscience refrains from unrestricted behavior due to lack of control. An example of natural behavior not amenable to controlled experiments is play.

Social play is involved in the development of adaptive behavior and prepares animals to deal with uncertainty in novel and social situations as adults.[2,3] Disruptions in social play are associated with cognitive and social developmental problems in rodents[2,4] and humans.[3,5] A brain area associated with social play is the prefrontal cortex (PFC), and this area is also enlarged in apes, which are among the most playful mammals.[6] Although cognitive neuroscience notes the importance of the PFC in the development of human social cognitive skills,[7–9] most studies investigating rodent PFC have focused on ethologically sterile, albeit tightly controlled, behavioral paradigms. Correlates of multiple variables have been identified in the PFC activity of overtrained animals. Yet, in the lab, rodents without a cortex survive and can learn to perform numerous behaviors.[10–12] Generally, the PFC is implicated in a range of functions, such as context representation,[13] event prediction,[14] decision-making,[15] adaptive behavior,[16] action monitoring,[17,18] and rule learning.[19]

Studying the neural activity of unconstrained behavior, like play, is a challenge. However, neuroscience is advancing quickly by applying statistical methods to the neural data of freely moving animals.[20–22] Such methods uncover regularity from the neural data in ways naive to behavioral recording. These provide a great tool to study the brain under agency-affording paradigms[23] and might help us understand the brain during natural behavior. Recently, these methods have been used in simple behavioral paradigms,[22] but extracting the most value out of them requires their implementation under free and complex behavior.

Our previous study[23] showed that rats can play a simple version of hide-and-seek with a human, and we later extended this study to pairs of rats observing each other play hide-and-seek.[24] In both

 © 2022 The Authors. Published by Elsevier Inc.
This is an open access article under the CC BY license (http://creativecommons.org/licenses/by/4.0/).

CellPress
OPEN ACCESS

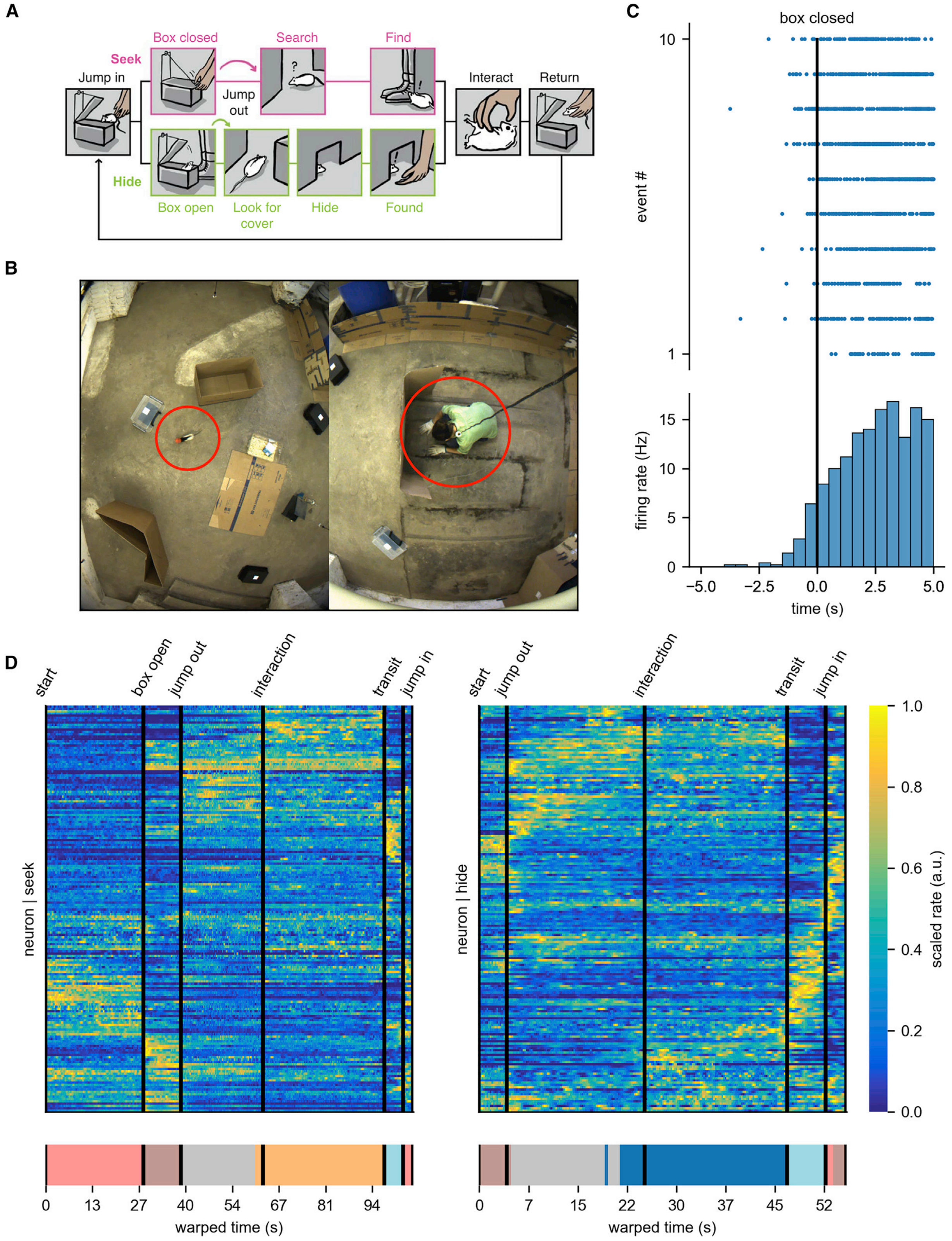

(legend on next page)

studies, we recorded the PFC of rats using tetrodes and a wireless system. In this paper, we use these datasets, attempting an inverse physiology approach to study the neural basis of this behavior and to probe methods of unsupervised population analysis during naturalistic behaviors.

We applied graphical state space models to segment neural data into behaviorally relevant epochs,[20] assuming that neural activity arises from switching between a finite number of brain states. We ask: (1) Can states be discovered in prefrontal cortical activity? (2) Do states correlate with behavior? (3) Does this approach allow the discovery of novel neural correlates of behavior?

We find that naively inferred states are related to hide-and-seek behaviors and accelerate the discovery of events and behaviors relevant for the PFC.

RESULTS

In Reinhold et al.,[23] four rats were trained to play hide-and-seek with humans (Figure 1A). Rats acquired the game quickly and learned to play both roles in a 30 m^2 room (Figure 1B). In hide-and-seek, the rats performed two types of trials, labeled after which role the rat was playing in the game.

While the game of hide-and-seek is a more free and less controlled form of behavior than what is usual for neuroscience experiments, the game structure (Figure 1A) allows us to define some time points in every trial that signal the phases of the game. We will refer to these throughout the paper. These are: (1) start, a time point indicating the start of a new trial; (2) box open (only in “seek” trials), the experimenter is hiding and opens the start-box remotely; (3) jump out, the rat jumps out of the start-box to either hide or seek; (4) interaction, the experimenter initiates interaction with the animal; (5) return transit, the animal is picked up to be returned to the start-box; (6) jump in, the animal jumps from the experimenter’s hand into the start-box; and (7) end, a time point after “jump in.”

We analyzed data of PFC neurons recorded with a wire-free neural logger (Deuteron Tech) while the animals played the game (N = 4, 13 sessions). As shown in our previous work, neurons responded to events in the game, such as the cell in Figure 1C responding to closing the start-box—the main cue differentiating hide from seek. Our previous analysis[23] pooled neurons from different sessions and averaged across trials, revealing clusters of cells whose mean activity is related to game events. Similarities in averaged activity (Figure 1D) of neurons from different sessions suggest that the population’s activity might be governed by a lower number of underlying factors. Recording subsets of neurons of the “same” population in individual sessions therefore means that we are looking at an underlying population activity “through different windows.”

First, we qualitatively replicate a part of the analysis from Reinhold et al.[23]

Stretching every trial to the same length while aligning them on the time points of the game, and ordering neurons based on their mean activity, reveals trial-averaged neuronal activity related to hide-and-seek game phases (Figure 1D, top).

Clustering mean activity not by neurons but by time (K-means clustering,[25] K = 6), highlights that there are distinct patterns of population activity at different stages of the game (clusters in colored bar, bottom plot of Figure 1D). This shows that—at this resolution—trial-averaged rates in game phases such as “transit” are assigned to the same cluster in both “seek” and “hide,” meaning that activity in these stages is similar across roles, while others show differences.

However, averaging gets rid of information encoded in trial-specific neural fluctuations, especially in a setting where trials have different durations and the animal’s behavior is free, unlike in traditional experiments. Motivated by the fact that simultaneous analysis of all recorded neurons might offer the statistical power needed for single-trial analysis,[26] we apply unsupervised state space models to the same neural data in a trial-by-trial manner. Doing so might reveal features of population activity correlated to behaviors not present in every trial or not directly related to documented external events.

Mutual information between hidden Markov model states and game phases

We adopt the neural population view[27] and analyze the joint activity of all simultaneously recorded PFC neurons. Neural activity at each time point is described by a vector of firing rates of the population’s neurons in the so-called “neural state space,” and—because of correlations between neurons—can be summarized by a set of latent factors.[28] The assumption underlying our analysis is that activity vectors corresponding to engaging in the same behavior on different occasions should be close in state space, and thus we can define a finite set of discrete states, corresponding to clusters of similar activity vectors, that make up the full set of activity vectors in a session. This assumption is illustrated by a generative model of neural activity in Figure 2A. In our analysis, at each time point we look at which discrete state the current population activity vector belongs to. Post hoc, we can investigate how these states relate to the animal’s behavior. We illustrate our approach on a single trial, on all trials of a session, and finally show that similar results are obtained in multiple sessions and datasets.

For each trial, first we bin each neuron’s spike times (250 ms) (Figure 2B, top), then preprocess using Gaussian-process factor analysis[29] (GPFA), identifying smoothly varying lower-dimensional signals underlying the population’s activity; the latent factors (Figure 2B, middle). Finally, we use a hidden Markov model (HMM)[30] fitted to the latent factors in all trials of a session to infer the most likely sequence of discrete states assumed to have generated the activity observed in the trial (Figure 2B, bottom).

Figure 1. Experimental setup of the hide-and-seek paradigm
(A) Structure of the game (from Reinhold et al.[23]).
(B) Experimental room.
(C) Spiking activity of a neuron aligned to the “box closed” time point.
(D) Stretched, trial-averaged, and normalized firing rates of all neurons (n = 176) pooled across sessions (n = 13, top). Neurons are ordered by hierarchical clustering for visualization. Clustering applied to the time points (K-means, K = 6, bottom).

CellPress
OPEN ACCESS

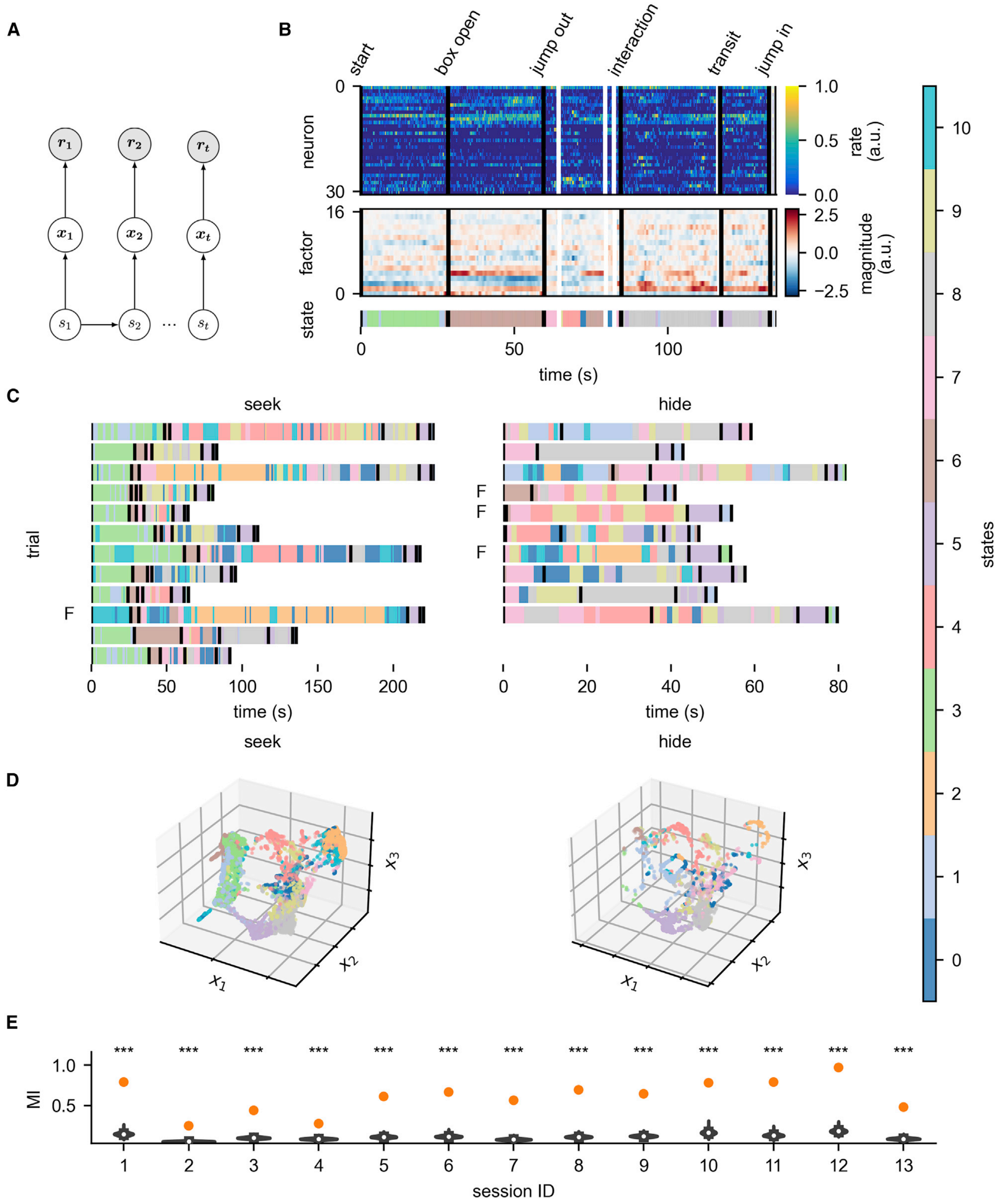

Figure 2. HMM state transitions coincide with events in the game

(A) Generative model of population activity. At each point in time PFC is in one of K discrete states (s_t) that follow each other with Markovian dynamics (bottom). Each discrete state has stereotypical vectors of latent factors (x_t, middle) associated with it, which are then assumed to underly the firing rate (r_t) of the population's neurons (top).

(legend continued on next page)

To visualize the segmentation, we uniquely color each discrete hidden state and plot which state was most likely active at every time point. Superimposing the time points at which the experimenters tagged an event based on the videos (Video S1)—the events signaling game phases (Figure 1A) in black and additional behaviors such as hiding, darting, and exploring in white—shows that switches in the inferred states tend to coincide with game events, as tagged from the video (see Figure 2B, vertical lines).

Applying the same procedure to every trial of a session (Figure 2C) shows a clear consistency in the order and appearance of the different latent states. This is true even though each trial's length, as well as the animal's behavior, is substantially different across trials. This observation led us to suspect that the states might be related to the specific actions, mental states, and behavioral contexts of the animal. Note that the model does not have any information about the behavioral events or the role the animal is playing; it relies on the neural data alone.

It is worth pointing out that the neural states identified in this session last many seconds and their duration has some variance (Figure S1A), which might point to the PFC encoding longer states than is generally assumed. This result is likely biased by our chosen timescale and the details of our analysis methods, and analysis on different levels of description could yield complementary insights.

How different discrete states correspond to clusters in neural state space is illustrated using uniform manifold approximation and projection (UMAP)[31] embeddings of population activity into 3 dimensions. Looking at the neural data in both "hide" and "seek" trials (Figure 2D), we see some clear differences and similarities in the use of states between the roles. Some states are shared, while others are more prominent in one role (Figures S1B–S1E). Note how state #3, which is predominantly active when the rat is inside the closed start-box, is mostly missing in "hide" trials and only present after the rat has jumped into the start-box at the end of trials.

To confirm our observations, we quantify the correspondence between HMM states and tagged behavioral epochs. We used all time points the experimenters had tagged to divide the trials into behavioral segments and calculated—using mutual information (MI)—the similarity between the behavioral and HMM segmentations. Figure 2E shows the MI for the HMM in each session (orange dot), compared with a distribution of randomly generated clusters (blue, see STAR Methods). The HMM segmentation based on neural data coincides with the behavioral segmentation above chance ($p < 0.0001$ in all sessions). The same analysis was also performed in a cross-validated manner (Figures S2A and S2B; see STAR Methods), and significance persists through a circular shuffling procedure (Figures S2C and S2D) as well. These results imply that, without prior information about behavior and structure of the game, clustering PFC population activity in time reflects game phases and behaviors identified by Reinhold et al.[23] This correspondence was confirmed using a random forest classifier on the GPFA factors, showing decoding of the behavioral state beyond chance (Figure S2E). Finally, decoding performance increases with the number of recorded neurons (Figure S2F) and is correlated with the MI score (Figure S2G).

Consistent representation of game phases and tagged behaviors

Our results hint at inferred states being active at certain game phases, consistently across trials. This shows—without averaging—the presence of distinct neural activity patterns in these periods.

To visualize this, we time-warped the states of each trial as we did to firing rates in Figure 1D (see STAR Methods). This aligns all trials of a session to each other, to highlight states which are locked to the phases of hide-and-seek and appear in a given phase on each trial (Figure 3A).

This holds for other sessions (Figure 3B), even though different neurons from different animals are recorded in them, suggesting that it is not specific to the small sample of neurons picked up by the recordings. However, because disjoint sets of neurons are recorded in every session, we need to fit a different HMM to each session. Given that labels are assigned randomly to the states during fitting, there is no one-to-one correspondence between states identified in different sessions. To reveal states related to the game phases across sessions, we attempted to match their states to each other. We use pairwise correlations of state probabilities through time as a similarity measure and match every session's states to a reference session (for details, see STAR Methods). Two examples of this matching procedure for "seek" are shown in Figure 3B, where re-coloring shows that states which are consistently locked to game phases are successfully matched to the reference session.

After matching every session's states to the reference, averaging state probabilities across all sessions (Figure 3C) reveals there are states which consistently occur at the same game-phase across sessions, even though they were identified on single trials of different sessions and/or animals instead of trial-averaged activity pooling neurons across sessions.

While stretching trials in length and aligning them identifies states that are locked to game phases, if our assumption that there is a correspondence between the animal's state in the world and the inferred brain states is correct—because hide-and-seek is so unrestricted—we could find states corresponding to behaviors or internal states not locked to the game.

(B) Analysis pipeline on a single trial. Binned spike counts (top) are projected to a lower number of latent factors using GPFA (middle), then segmented into discrete neural states using HMM (bottom). Black vertical lines show time points signaling the game phases, white lines are non-phase-locked events (entering a box, darting).
(C) Inferred discrete states in all trials of a session (session #12) show shared structure in the states across trials. "F" markings indicate failed trials. Black vertical lines indicate the game phases.
(D) 3D UMAP embedding of the latent factors, colored by states (session #12).
(E) Mutual information of behavioral and HMM states (orange points) are above chance ($p < 0.0001$, distribution of scores by random clustering in blue) in all sessions. Star-code shows the p value for the bootstrap, *** = 0.001.
See Figures S1 and S2 and Video S1.

CellPress
OPEN ACCESS

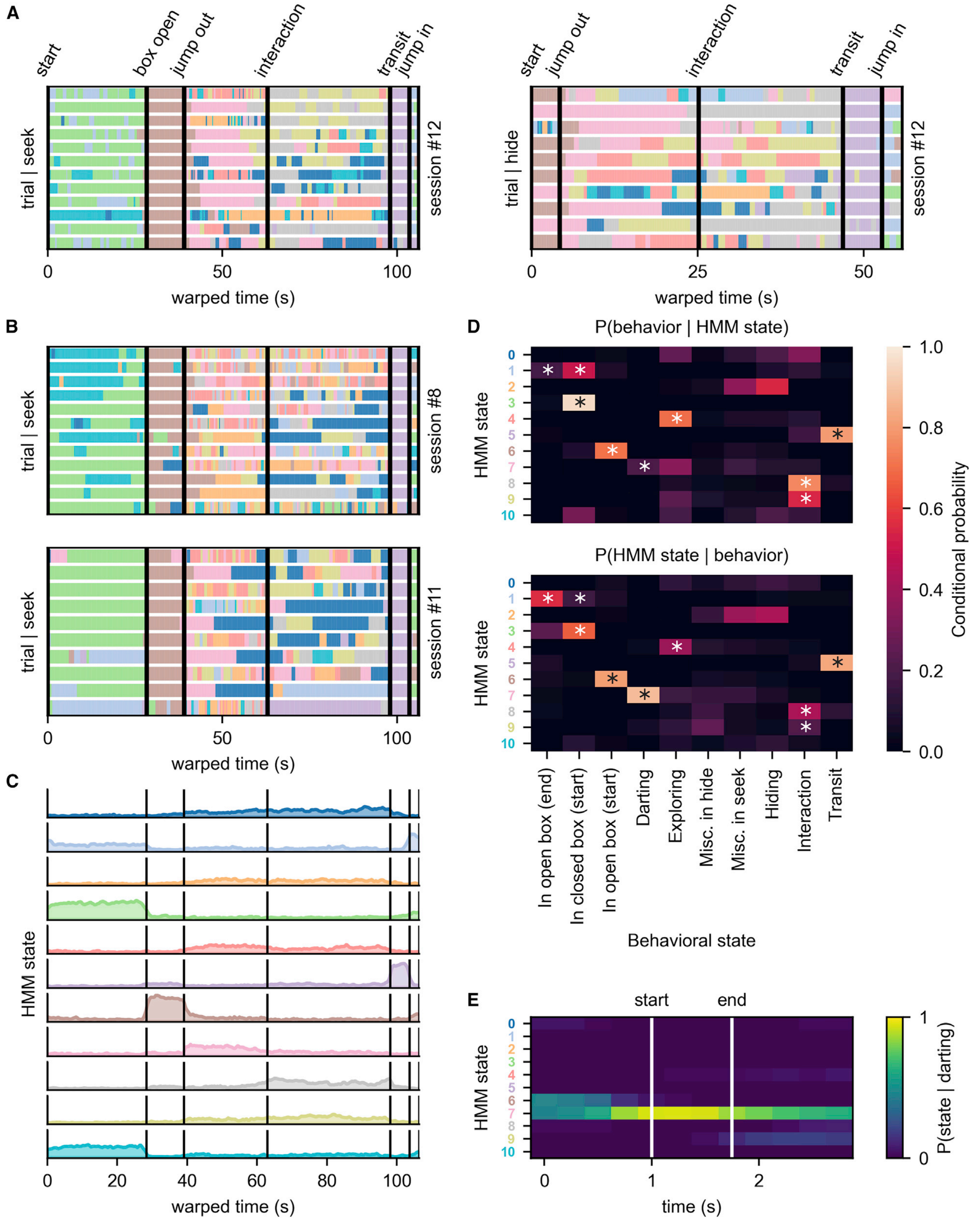

Figure 3. States related to the hide-and-seek game are identified across sessions

(A) Hidden states of all trials are matched to the same length and aligned on the time points signaling the game phases (black).

(B) Stretched states in two example sessions matched to the reference. Session #8 is a different animal, and session #11 is the same animal as the reference.

(legend continued on next page)

To start, we look at co-occurrences of the HMM states with behavioral states of the animal (tagged based on the videos), including behaviors not locked to game phases.

Figure 3D shows conditional probabilities, P(HMM state|behavioral state) and P(behavioral state|HMM state), to illustrate this point. Rows of P(behavioral state|HMM state) (Figure 3D, top) show what behavior the animal was most likely engaged in when a state was active, while columns of P(HMM state|behavioral state) (Figure 3D, bottom) show which states were mostly active during a tagged behavior. Using a bootstrap analysis, we find statistically significant correspondences between HMM and behavioral states, denoted by stars on Figure 3D ($p < 0.05$ post Holm-Bonferroni correction). Performing the same significance test on all sessions independently (Figure S3) shows the number of sessions having an HMM state significantly associated with a behavior, showing the repeated appearance of game-related states.

One behavioral state not locked to game phases is darting, which is when the animal gallops forward on a straight path with high velocity, and can happen at different game phases. From P(HMM state|behavioral state = darting) (column in the bottom panel of Figure 3D corresponding to "darting"), it is apparent that there is a single inferred state, state #7, which is most likely active during this behavior.

Investigating the relationship between state #7 and darting, we extracted the most likely states during every darting event, with 1 s before and after. The probability of each state across all darting events (Figure 3E) illustrates the correspondence between state #7 and darting. Despite this correspondence, the relationship is not one-to-one. Looking at P(behavioral state|HMM state = 7) (row in top panel of Figure 3D corresponding to state #7) reveals that this state is not entirely specific to epochs identified by the experimenters as darting.

Generally, our data analysis is filtered by experimenters' decisions to tag or not to tag behaviors. This limitation is external to the HMM, which infers states in the brain without behavioral bias. Next, we used this feature to accelerate the discovery of novel, behaviorally relevant states.

HMM segmentation discovers novel neurally relevant behavior

Correlates of tagged behaviors emerge from neural states inferred from PFC activity alone. However, our approach's strength lies in identifying what is represented in neural activity in an unsupervised, bottom-up way. It is possible that there are states that represent parts of the environment or behaviors not anticipated as relevant, defining a brain-based ethogram.

To unbiasedly discover what states represent, for each HMM state we extracted videoclips where that state was active. This is related to what is done in other studies segmenting behavioral data.[32–34] It is, however, fundamentally different in that we are not segmenting the behavior in the videos based on behavioral data, but segmenting videos based on the neural data alone. This is reverse physiology and comparable to doing a spike-triggered average, in this case a "state-triggered collection" of videoclips.

Firstly, we confirm that states earlier identified as being related to game phases are also apparent when looking at video collections. Even if we had no prior information that there were game phases, it would be discoverable from the extracted collections that, e.g., state #5 is active during transit to the start-box (Figure 4A; Video S2). Figure 4A showcases a compression of the information gathered from videos in a single figure. Each time the state is active (Figure 4A, left), we extract image regions of interest that deviate from a background (i.e., we extract changes from the background), combined and superimposed into a single image on a background frame (Figure 4A, right; see STAR Methods), producing a simultaneous graphical representation of the behaviors during a neural state. Figure 4A shows how the "transit state" being active corresponds to when the rat was on the experimenter and transported to the start-box, capturing paths taken by the experimenter back to the start-box in 3 different sessions. This is not a prediction of behavior, just an empirical characterization of what happened at the same time as the brain was in this state. This shows that a reverse physiology approach may confirm our pre hoc assumptions, but the same approach could lead to the discovery of unassumed descriptions for what the brain is encoding.

Extending this methodology to each state, we describe what happened in the experiment when that state was active. This can be seen as a brain-based ethogram, naively analyzing what elicits state changes in the PFC without looking at the behavior first. To exemplify, we look at the reference session (session #12).

As mentioned before, tagged behaviors cannot be considered ground truth. We found that state #7—shown to dominate during darting (Figure 3E)—is predominantly active when the animal is running, and the short darting epochs identified by the subjective criteria of the experimenters are a subset of this behavior (Figure 4B; Video S3). An approximation of the animal's speed when each state was active shows that—apart from the transit state when transported by the human experimenter—the animal was moving fastest when state #7 was active (Figures S4A and S4B).

Consistent with Figure 3C, we found a state associated with the proximity of the experimenter mostly active during interaction and play. State #8 is active during play with the experimenter and is sometimes activated upon their approach (Video S4; Figure S5A). This state appears absent in failed trials where the play reward is omitted (Figure S5B).

To this point, we have shown bottom-up discovery of states for which we had pre hoc expectations. However, this method resulted in surprising discoveries as well. For example, two states are active when the rat is walking alongside walls (#9 and #4) (Figure 4C; Video S5, quantified in Figures S4C and

(C) Probability of state occurrence through time (averaged across all sessions ($n = 13$) from all rats ($N = 4$) after matching to the reference).
(D) Conditional probabilities P(behavioral state|HMM state) and P(HMM state|behavioral state) reveal states associated with behaviors not necessarily locked to game phases. Stars represent HMM-behavioral state pairs that have significant conditional probability ($p < 0.05$, Holm-Bonferroni correction, based on bootstrap analysis).
(E) Probability of each state occurring during darting events (stretched for visualization) shows that state #7 is associated with darting.
See also Figure S3.

CellPress
OPEN ACCESS

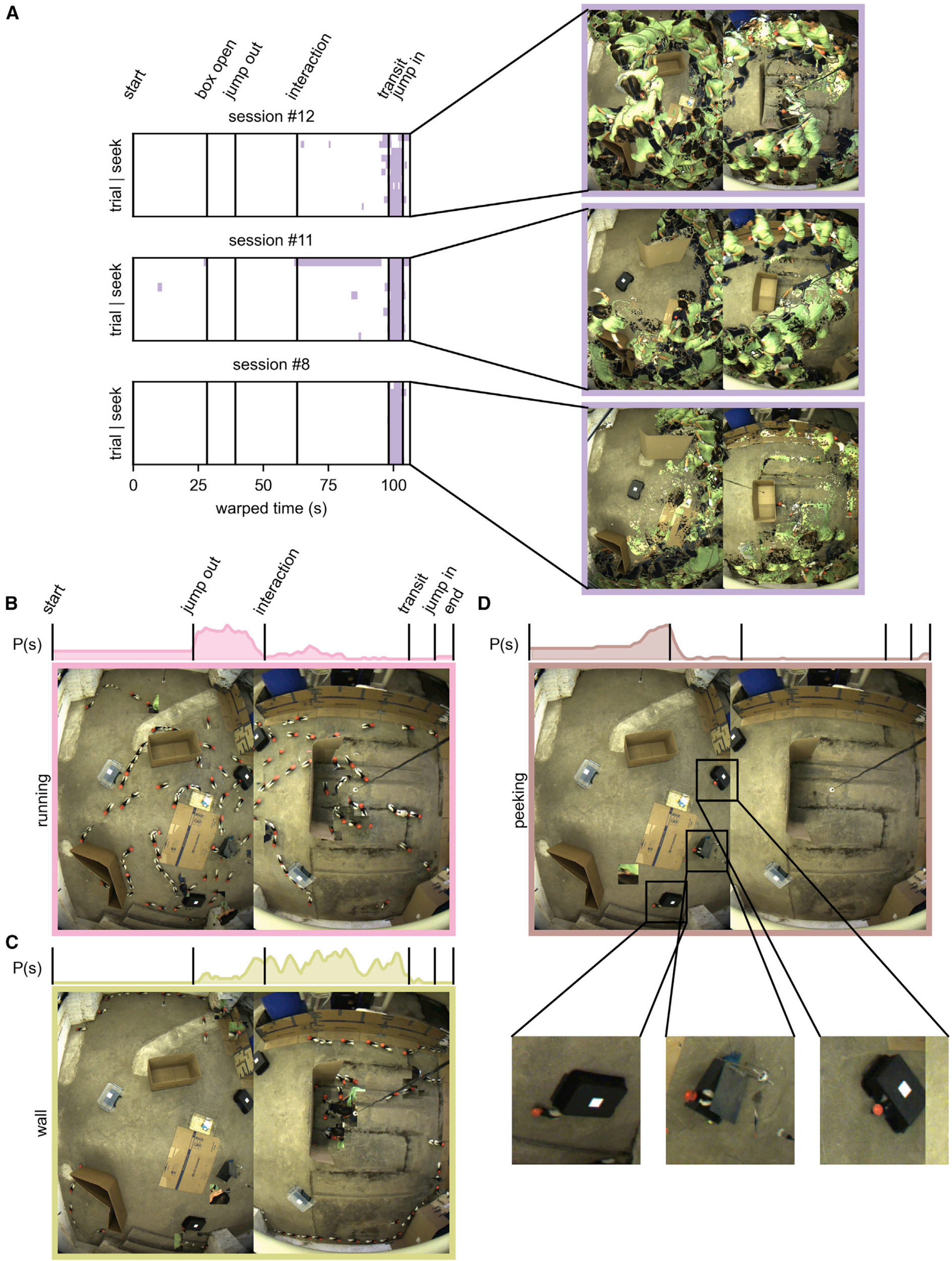

(legend on next page)

S4D). Anecdotally, one state seems related to slowly walking along the wall, while another presents wall exploration.

Even though tagging rest periods as meaningful behavior a priori is uncommon, our reverse physiology of session #12 showed states consistently active when the rat seems to be resting and inactive. The apparent examples of these states are the long periods in two of the "seek" trials with switches between states #2 and #10 (see Figure 2C). Extracting the videos, we found that these correspond to the animal stopping behind a cardboard wall and not looking for the experimenter. These states are generally active when the animal is resting or seems unengaged by the task (see speeds in Figure S4A) and are notably present in the failed "seek" trial (Figure S5B). We do not notice apparent behavioral differences from our collections between both states. We note, however, that state #2 includes longer states (Figure S1A), and state #10 sometimes activates when the animal waits in the start-box. Differences picked up by the HMM between state #2 and #10 could be beyond our behavioral resolution or related to processes such as attention or planning.

Some inferred states relate to the animal's interaction with experimental boxes. States #3 and #1 are active when the rat is in or jumping into the start-box. Additionally, state #1 is sometimes active when the rat is in a hiding box. One very interesting state (#6) activates when the rat is in the opened start-box. When extracting videos for the state unbiasedly, we noticed the same state is active when the rat peeks out of hiding boxes in the play arena (Figure 4D, insets; Video S6).

Overall, we find that hidden states, identified purely on neural data, reflect the behavioral state, game phase, and actions of the animal. We wondered whether, while observing the game, the PFC could recover states related to the behavior of another rat playing hide-and-seek.

HMM states differ between observing and playing behaviors

To test whether neural states in the PFC mirror states of another rat, we applied the same method to a dataset that compares playing hide-and-seek to observing hide-and-seek.[24] Rats ($N = 4$, 12 sessions) were recorded while playing hide-and-seek with the experimenter and while observing another rat play the game from inside a glass box. Previous work[24] showed that cells during observing have lower firing rates and are not responsive to the play going on with the other rat. We re-analyzed this data using our bottom-up framework.

We increased the possible states ($K = 18$), accounting for states that might occur when the animal is observing. State analysis confirms single-cell analysis,[24] in that the activity in the PFC during observing and playing is starkly different. Our previous analysis was event-related, i.e., biased by the experimenter. The model, however, without bias, infers one set of states active during playing and another distinct set for observing (Figure 5A). States during play (Figure 5A, top) hold a relation to game phases, reaffirming our previous findings.

Indeed, the conditional probability of a trial being an observing one given the active state clearly splits the set of states in most sessions (Figures 5B and S6; see STAR Methods). Embedding neural activity into 3 dimensions using UMAP and coloring by HMM state (Figure 5C) demonstrates the remarkable separation between playing and observing in neural state space. Additionally, it shows how HMM states correspond to clusters in this space.

MI on playing trials (Figures 6A and 6D) reproduces our previous findings ($p \leq 0.001$ in all sessions). Figure 6B shows the analysis applied to neural data in the observing rat, comparing HMM states with its own tagged behavior (grooming, engaged, or resting; see STAR Methods). Although the correspondence is less clear, the HMM is picking up meaningful states for the observing rat during observing trials (Figure 6B, $p < 0.05$ in 8 sessions). Finally, HMM segmentation of activity in the observing rat does not show a significant relationship with the manual segmentation of the other (observed) rat's playing behavior (Figure 6C, $p < 0.05$ in 2 sessions only).

Even though two sessions have significant MI between the HMM states while observing and the other rat's play, and in some sessions there are states co-occurring in observing and playing trials, we found no state mirroring of play. States with a conditional probability P(observing|state) between 0.3 and 0.7 are rare (median: 12.2% of total time). With few neurons it is hard to determine whether these infrequent finds are due to sharing states or poor neural resolution to resolve them. What is clear, is the robust tendency to separate state representations in the two contexts and the value of the method to demonstrate these.

DISCUSSION

In contrast to traditional neuroscience, the hide-and-seek paradigm[23] allows rats to engage in a wide range of unrestricted behaviors. We show that, despite no two trials of hide-and-seek being the same, it is possible to meaningfully analyze neural activity, even with relatively few neurons. We here extend on previous work showcasing the power of state space analysis to uncover behaviorally relevant neural states.

We recognize and were influenced by work that applied akin statistical methods to neural data. Linderman et al.[20] conducted similar analyses on the simpler behavior of *C. elegans*. Rubin et al.[22] report similar findings from Ca^{2+} recordings in mice during much simpler behavior. Recanatesi et al.[35] have shown that HMMs fitted to neural activity in the secondary motor cortex of rats accurately predict sequences of self-initiated actions. We stipulate—like Rubin et al.[22]—that our method is useful for exploratory analysis and identifying aspects of an experiment and the animals' behavior that are reflected in the neural activity of a brain area whose function is yet unclear.

Figure 4. Discovery of new neurally relevant behaviors using video extraction
(A) Bottom-up analysis confirms the "transit neural state." Times when state #5 was active in 3 sessions (left, after state matching). Filtered video frames during these times superimposed on each other (right).
(B–D) Superimposing extracted video frames illustrates behavior associated with individual neural states. (B) Running (state #7), (C) walking along walls (state #9), and (D) peeking out of boxes (state #6). Marginal plots above: probability of state occurrence through time across trials of the session.
See also Figures S4 and S5 and Videos S2, S3, S4, S5, and S6.

CellPress
OPEN ACCESS

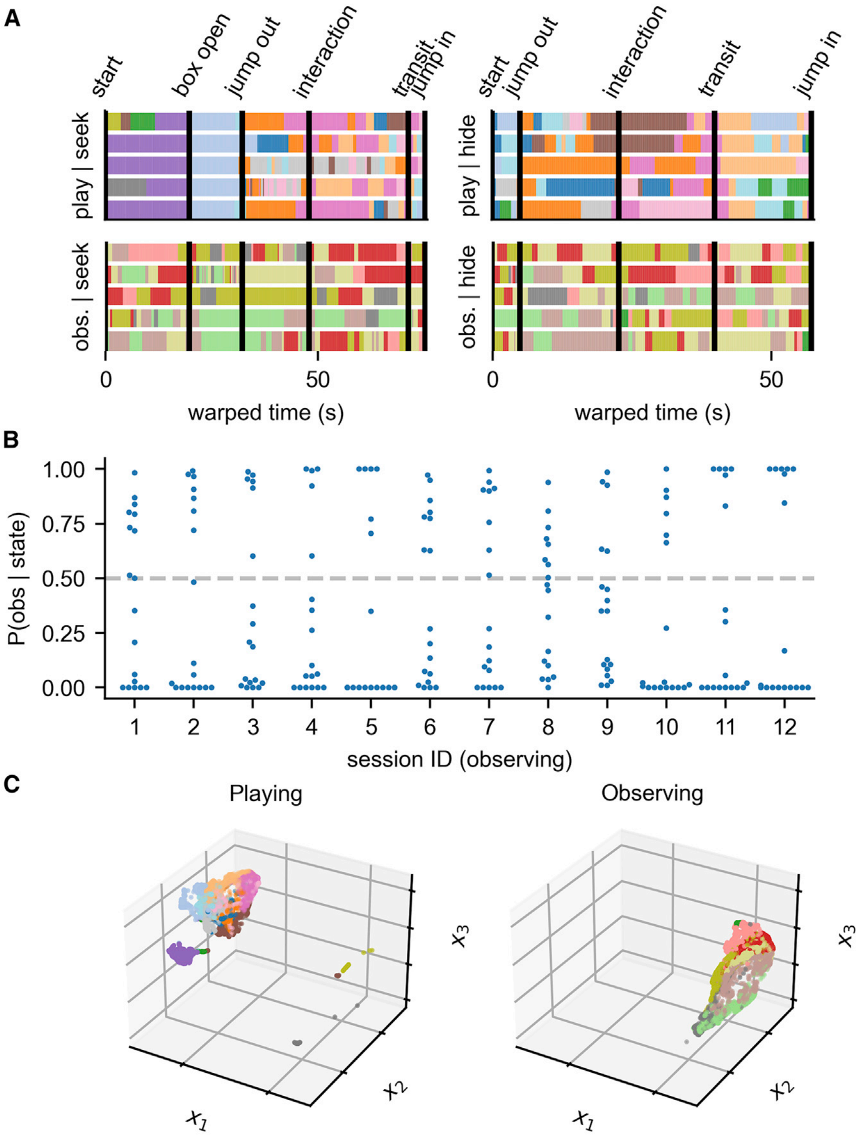

Figure 5. Playing and observing hide-and-seek are associated with distinct sets of neural states

(A) Stretched HMM states on each trial of a session show the game structure when playing the game, as well as another set of states (with no connection to game phases) when observing.

(B) P(observing|neural state) between playing and observing neural states. (Each dot corresponds to a state.)

(C) 3D UMAP embedding in an example session illustrates the separation between playing and observing states.

In our study, we recorded the simultaneous activity of a maximum of 31 neurons. We find the fact that we were able to read out the animals' approximate behavioral states—based on the HMM states as well as using supervised decoding—from few neurons noteworthy. Our findings confirm previous reports[13,17,18,22] of PFC function, taken to higher behavioral complexity and freedom. Our approach is similar to Recanatesi et al.[35] and Rubin et al.,[22] the important advancement being that hide-and-seek is a vastly more complex behavior than previously studied. We hypothesize that our approach's success is related to the functions of the PFC and are hopeful that larger-scale recordings combined with similar analyses might provide a more comprehensive view into the PFC during complex behavior(s). Additionally, we constrained ourselves to fitting models with an equal number of states to different sessions; yet, this approach can be applied in a more flexible way, where the number of states can vary across sessions, days, or animals. Furthermore, though unaddressed here, detailed structure-function work with high density probes should clarify the difference in role, if any, between sub-regions of the rat PFC, applying models to different areas.

The segmentation by the HMM is perfectible. Sometimes a "single behavioral state" (such as inside the closed box) is partitioned into multiple states. These longer states (many seconds long) might be better described by models switching between different linear or non-linear dynamics. Such models have been used by Linderman et al.,[20] inferring segmentations of worm behavior that closely matches that of human experts. Our results are in line with the findings of Linderman et al.,[20] that for segmentation alone, simpler models are enough.

Future work should not overlook the quantification of the animals' behavior. Even though the original experiment was not technically designed for a detailed behavioral analysis, we approximated relations between some behavioral variables and latent states. We speculate that adding more hidden states to our models might lead to a segmentation on a finer scale, but our behavioral data are not detailed enough (e.g., no pose information) for a meaningful analysis on that timescale. As a supplementary to manual segmentation of behavior, Wiltschko et al.[34] perform unsupervised segmentation like ours directly on the behavior of mice, using autoregressive (AR)-HMMs decomposing mice's pose dynamics into behavioral syllables. Markowitz et al.[36] use segmentation tools developed by Wiltschko et al.[34] to relate activity in striatum to "behavioral syllables" in mice, and stipulate that switches between the behavioral modules might be controlled by higher-order brain areas, such as the PFC. Batty et al.[32] also use AR-HMMs to segment behavioral videos, producing a lower-dimensional representation of behavior and aiding the decoding of neural activity.

Unfortunately, our data had no one-to-one correspondence between the neurons recorded in the different sessions, leading to us having to fit separate HMMs per session. As detailed by Dabagia et al.,[37] an interesting idea for combining data from multiple recordings—assuming the structure of neural activity

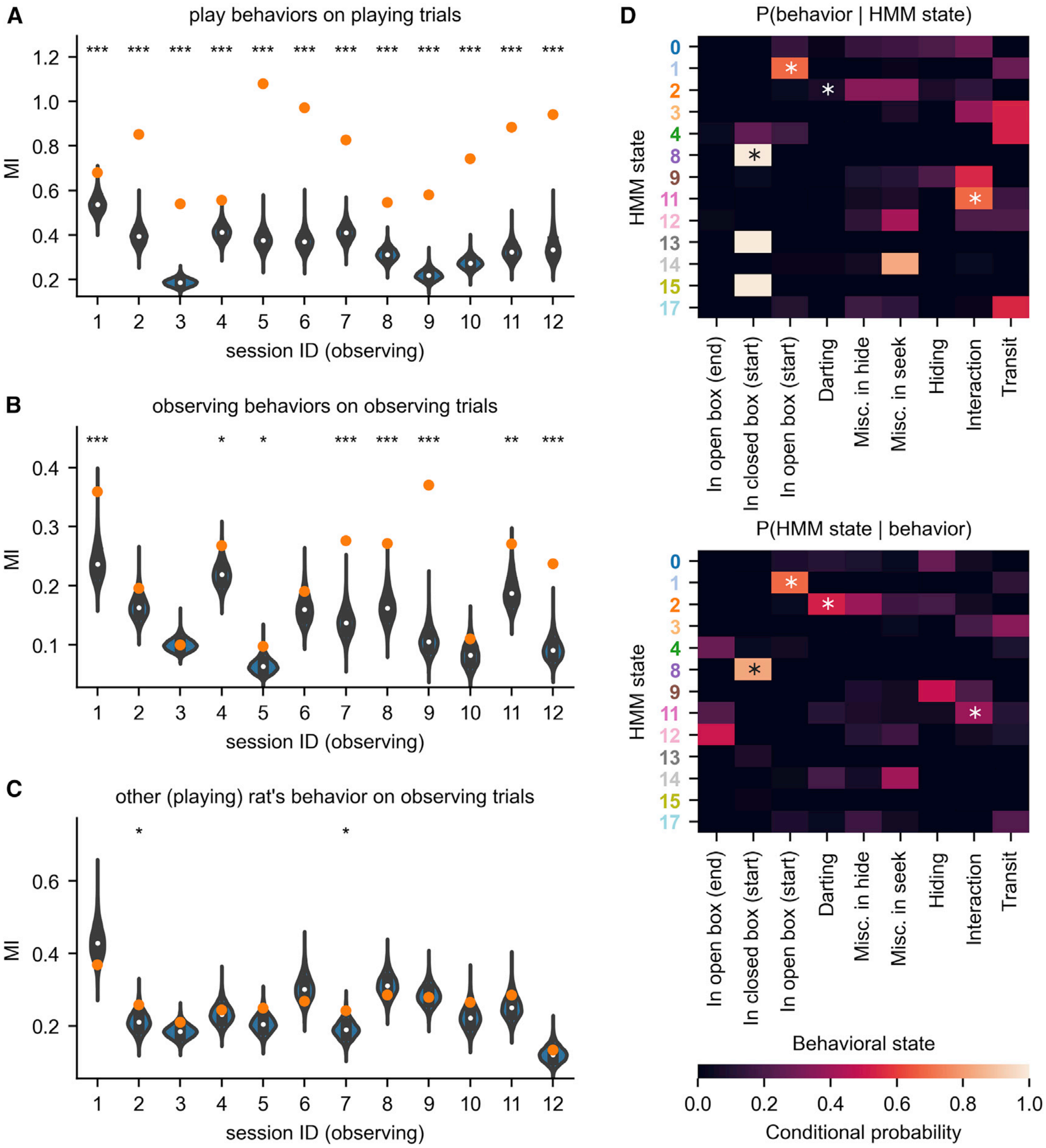

Figure 6. Results about playing are reproduced in a second dataset, but state mirroring is absent

(A) Mutual information of HMM states of the playing rat with its own play behavior is significant.

(B) Mutual information of HMM states of the observing rat with its own tagged observing behavior (see STAR Methods) shows significance in a different context.

(C) There is less significant mutual information between inferred HMM states of the observing rat with the manual segmentation of the playing rat's behavior.

(D) Similar to Figure 3D, conditional probabilities P(behavioral state|HMM state) and P(HMM state|behavioral state) reveal significant correspondence between certain HMM-behavioral state pairs (stars represent $p < 0.05$, Holm-Bonferroni correction). The star-code in (A)–(D) shows the p value for the bootstrap with * = 0.05, ** = 0.01, *** = 0.001.

See also Figure S6.

remains stable[38]—could be to align the distributions of population activity in them. Perhaps high-yield recordings allow for a better estimate of the structure of population activity, making alignments feasible and permitting joint analysis from different sessions and individuals.

Despite all these challenges, we have used HMMs to naively detect meaningful behaviors that resulted in state changes in the PFC. We have contributed to showing the robust differences in state representation between play and observing play, pointing toward the value of studying the PFC in different arousal states. Combining these methods with experiments where animals engage in many dissimilar tasks, behaviors, and contexts would contribute to understanding the PFC.

Overall, our work shows that a few simultaneously recorded neurons from the PFC are enough to decode the hide-and-seek game phases in a "state-by-state" manner. The ease with which a simple HMM allows us to infer such changes in behavior signifies the clear role of the PFC in representing behavioral states in naturalistic and complex conditions. These conclusions are in line with work demonstrating the PFC's role in the representation of current rules[16] and its putative role in computing belief states.[39] Such a representation of state is an important piece of the puzzle in the reinforcement learning framework. Overall, it is apparent that HMM neural state analyses expand our ability to relate multi-neuron activity to behavior, and hence this type of analysis addresses a fundamental issue in neuroscience. Our analysis of the observing-playing dataset uses a novel approach to address the question of "mirroring" from a state perspective ("mirror states") during observing play behavior in the PFC. We think our brain-state-driven approach could in the future be implemented in more controlled tickling experiments where there is evidence of behavioral contagion and neural mirroring in the somatosensory cortex.[40] The combination of reverse physiology with neural state analyses will also undoubtedly play a future role in the neuroscience of naturalistic behavior. Specifically, recent advances in recording technologies[41] applied to freely living animals would help to clarify the role of PFC in action selection and execution of an animal's complete ethogram.

STAR★METHODS

Detailed methods are provided in the online version of this paper and include the following:

- KEY RESOURCES TABLE
- RESOURCE AVAILABILITY
  - Lead contact
  - Materials availability
  - Data and code availability
- EXPERIMENTAL MODEL AND SUBJECT DETAILS
- METHOD DETAILS
  - Video recordings
  - Neural acquisition
- QUANTIFICATION AND STATISTICAL ANALYSIS
  - Selecting sessions for analysis
  - Segmenting neural data in time
  - Visualization using UMAP
  - Stretching
  - Mutual information
  - Decoding analysis
  - Matching
  - Video summary figures
  - Software tools

SUPPLEMENTAL INFORMATION

Supplemental information can be found online at https://doi.org/10.1016/j.cub.2022.04.068.

ACKNOWLEDGMENTS

Funded by the Einsteinstiftung (ESB project "Dynamics of electronically coupled neuronal networks" A-2016-350) and the ERC synergy grant BrainPlay. We thank Annika Reinhold, Konstantin Hartmann, and Miguel Concha, who with J.I.S.-S. collected the data analyzed, and Caroline Hu (Hudrewthis) for the graphical abstract design. B.B. was supported by the German Academic Exchange Service (DAAD). J.I.S.-S. is currently supported by a Human Frontier Science Program Long Term Postdoctoral Fellowship (HFSP Ref. LT000289/2021-L).

AUTHOR CONTRIBUTIONS

M.B. and J.I.S.-S. provided the data. B.B. implemented the analysis. B.B., M.B., and J.I.S.-S. analyzed the data. B.B. and J.I.S.-S. designed the analysis and made the figures. B.B., M.B., and J.I.S.-S. conceptualized the study. B.B., M.B., and J.I.S.-S. wrote the paper. M.B. provided funds for the study.

DECLARATION OF INTERESTS

The authors declare no competing interests.

Received: September 28, 2021
Revised: March 29, 2022
Accepted: April 22, 2022
Published: May 18, 2022

REFERENCES

1. Tinbergen, N. (1951). The Study of Instinct (Clarendon Press/Oxford University Press).
2. Siviy, S.M. (2016). A brain motivated to play: insights into the neurobiology of playfulness. Behaviour *153*, 819–844.
3. Vanderschuren, L.J.M.J., Achterberg, E.J.M., and Trezza, V. (2016). The neurobiology of social play and its rewarding value in rats. Neurosci. Biobehav. Rev. *70*, 86–105.
4. Vanderschuren, L.J.M.J., and Trezza, V. (2013). What the Laboratory rat has taught us about social play behavior: role in behavioral development and neural mechanisms. In The Neurobiology of Childhood Current Topics in Behavioral Neurosciences, S.L. Andersen, and D.S. Pine, eds. (Springer), pp. 189–212.
5. Alessandri, S.M. (1992). Attention, play, and social behavior in ADHD preschoolers. J. Abnorm. Child Psychol. *20*, 289–302.
6. Smaers, J.B., Gómez-Robles, A., Parks, A.N., and Sherwood, C.C. (2017). Exceptional evolutionary expansion of prefrontal cortex in great apes and humans. Curr. Biol. *27*, 714–720.
7. Amodio, D.M., and Frith, C.D. (2006). Meeting of minds: the medial frontal cortex and social cognition. Nat. Rev. Neurosci. *7*, 268–277.
8. Anderson, S.W., Bechara, A., Damasio, H., Tranel, D., and Damasio, A.R. (1999). Impairment of social and moral behavior related to early damage in human prefrontal cortex. Nat. Neurosci. *2*, 1032–1037.
9. Grossmann, T. (2013). The role of medial prefrontal cortex in early social cognition. Front. Hum. Neurosci. *7*, 340.
10. Kawai, R., Markman, T., Poddar, R., Ko, R., Fantana, A.L., Dhawale, A.K., Kampff, A.R., and Ölveczky, B.P. (2015). Motor cortex is required for learning but not for executing a motor skill. Neuron *86*, 800–812.
11. Lopes, G., Nogueira, J., Paton, J.J., and Kampff, A.R. (2016). A robust role for motor cortex. Preprint at bioRxiv. https://doi.org/10.1101/058917.
12. Whishaw, I.Q. (1990). The decorticate rat. In The Cerebral Cortex of the Rat (The MIT Press), pp. 239–267.
13. Hyman, J.M., Ma, L., Balaguer-Ballester, E., Durstewitz, D., and Seamans, J.K. (2012). Contextual encoding by ensembles of medial prefrontal cortex neurons. Proc. Natl. Acad. Sci. USA *109*, 5086–5091.
14. Alexander, W.H., and Brown, J.W. (2014). A general role for medial prefrontal cortex in event prediction. Front. Comput. Neurosci. *8*, 69.
15. Euston, D.R., Gruber, A.J., and McNaughton, B.L. (2012). The role of medial prefrontal cortex in memory and decision making. Neuron *76*, 1057–1070.

16. Karlsson, M.P., Tervo, D.G.R., and Karpova, A.Y. (2012). Network resets in medial prefrontal cortex mark the onset of behavioral uncertainty. Science *338*, 135–139.

17. Lapish, C.C., Durstewitz, D., Chandler, L.J., and Seamans, J.K. (2008). Successful choice behavior is associated with distinct and coherent network states in anterior cingulate cortex. Proc. Natl. Acad. Sci. USA *105*, 11963–11968.

18. Balaguer-Ballester, E., Lapish, C.C., Seamans, J.K., and Durstewitz, D. (2011). Attracting dynamics of frontal cortex ensembles during memory-guided decision-making. PLoS Comput. Biol. *7*, e1002057.

19. Durstewitz, D., Vittoz, N.M., Floresco, S.B., and Seamans, J.K. (2010). Abrupt transitions between prefrontal neural ensemble states accompany behavioral transitions during rule learning. Neuron *66*, 438–448.

20. Linderman, S., Nichols, A., Blei, D., Zimmer, M., and Paninski, L. (2019). Hierarchical recurrent state space models reveal discrete and continuous dynamics of neural activity in *C. elegans*. Preprint at bioRxiv. https://doi.org/10.1101/621540.

21. Nassar, J., Linderman, S.W., Bugallo, M., and Park, I.M. (2019). Tree-structured recurrent switching linear dynamical systems for multi-scale modeling. Preprint at arXiv. arXiv:1811.12386.

22. Rubin, A., Sheintuch, L., Brande-Eilat, N., Pinchasof, O., Rechavi, Y., Geva, N., and Ziv, Y. (2019). Revealing neural correlates of behavior without behavioral measurements. Nat. Commun. *10*, 4745.

23. Reinhold, A.S., Sanguinetti-Scheck, J.I., Hartmann, K., and Brecht, M. (2019). Behavioral and neural correlates of hide-and-seek in rats. Science *365*, 1180–1183.

24. Concha-Miranda, M., Hartmann, K., Reinhold, A., Brecht, M., and Sanguinetti-Scheck, J.I. (2020). Play, but not observing play, engages rat medial prefrontal cortex. Eur. J. Neurosci. *52*, 4127–4138.

25. Macqueen, J. (1967). Some methods for classification and analysis of multivariate observations. In 5th Berkeley Symposium on Mathematical Statistics and Probability, pp. 281–297.

26. Cunningham, J.P., and Yu, B.M. (2014). Dimensionality reduction for large-scale neural recordings. Nat. Neurosci. *17*, 1500–1509.

27. Saxena, S., and Cunningham, J.P. (2019). Towards the neural population doctrine. Curr. Opin. Neurobiol. *55*, 103–111.

28. Gallego, J.A., Perich, M.G., Miller, L.E., and Solla, S.A. (2017). Neural manifolds for the control of movement. Neuron *94*, 978–984.

29. Yu, B.M., Cunningham, J.P., Santhanam, G., Ryu, S.I., Shenoy, K.V., and Sahani, M. (2009). Gaussian-process factor analysis for low-dimensional single-trial analysis of neural population activity. J. Neurophysiol. *102*, 614–635.

30. Rabiner, L.R. (1989). A tutorial on hidden Markov models and selected applications in speech recognition. Proc. IEEE *77*, 257–286.

31. McInnes, L., Healy, J., and Melville, J. (2020). UMAP: uniform manifold approximation and projection for dimension reduction. Preprint at axXiv. arXiv:1802.03426.

32. Batty, E., Whiteway, M., Saxena, S., Biderman, D., Abe, T., Musall, S., Gillis, W., Markowitz, J., Churchland, A., Cunningham, J.P., et al. (2019). BehaveNet: nonlinear embedding and Bayesian neural decoding of behavioral videos. In NIPS'19: Proceedings of the 33rd International Conference on Neural Information Processing Systems.

33. Johnson, M.J., Duvenaud, D., Wiltschko, A.B., Datta, S.R., and Adams, R.P. (2017). Composing graphical models with neural networks for structured representations and fast inference. Preprint at arXiv. arXiv:1603.06277.

34. Wiltschko, A.B., Johnson, M.J., Iurilli, G., Peterson, R.E., Katon, J.M., Pashkovski, S.L., Abraira, V.E., Adams, R.P., and Datta, S.R. (2015). Mapping sub-second structure in mouse behavior. Neuron *88*, 1121–1135.

35. Recanatesi, S., Pereira-Obilinovic, U., Murakami, M., Mainen, Z., and Mazzucato, L. (2022). Metastable attractors explain the variable timing of stable behavioral action sequences. Neuron *110*, 139–153.e9.

36. Markowitz, J.E., Gillis, W.F., Beron, C.C., Neufeld, S.Q., Robertson, K., Bhagat, N.D., Peterson, R.E., Peterson, E., Hyun, M., Linderman, S.W., et al. (2018). The striatum organizes 3D behavior via moment-to-moment action selection. Cell *174*, 44–58.e17.

37. Dabagia, M., Kording, K.P., and Dyer, E.L.. Comparing high-dimensional neural recordings by aligning their low-dimensional latent representations. Preprint at Semantic Scholar. https://www.semanticscholar.org/paper/Comparing-high-dimensional-neural-recordings-by-Dabagia-Kording/9d8b196c4786f9d70499c3a4638a1d706c39786f.

38. Gallego, J.A., Perich, M.G., Chowdhury, R.H., Solla, S.A., and Miller, L.E. (2020). Long-term stability of cortical population dynamics underlying consistent behavior. Nat. Neurosci. *23*, 260–270.

39. Gershman, S.J., and Uchida, N. (2019). Believing in dopamine. Nat. Rev. Neurosci. *20*, 703–714.

40. Kaufmann, L.V., Brecht, M., and Ishiyama, S. (2021). Tickle Contagion in the somatosensory cortex. Preprint at bioRxiv. https://doi.org/10.1101/2021.10.22.465426.

41. Jun, J.J., Steinmetz, N.A., Siegle, J.H., Denman, D.J., Bauza, M., Barbarits, B., Lee, A.K., Anastassiou, C.A., Andrei, A., Aydın, Ç., et al. (2017). Fully integrated silicon probes for high-density recording of neural activity. Nature *551*, 232–236.

42. Lopes, G., Bonacchi, N., Frazão, J., Neto, J.P., Atallah, B.V., Soares, S., Moreira, L., Matias, S., Itskov, P.M., Correia, P.A., et al. (2015). Bonsai: an event-based framework for processing and controlling data streams. Front. Neuroinform. *9*, 7.

43. Pachitariu, M., Steinmetz, N., Kadir, S., Carandini, M.,D., and H.K.. (2016). Kilosort: realtime spike-sorting for extracellular electrophysiology with hundreds of channels. Preprint at bioRxiv. https://doi.org/10.1101/061481.

44. Satopaa, V., Albrecht, J., Irwin, D., and Raghavan, B. (2011). Finding a "Kneedle" in a haystack: detecting knee points in system behavior. In 31st International Conference on Distributed Computing Systems Workshops, pp. 166–171.

45. Linderman, S., Antin, B., Zoltowski, D., and Glaser, J. (2020). SSM: Bayesian learning and inference for state space models https://github.com/lindermanlab/ssm.

46. Fox, E.B., Sudderth, E.B., Jordan, M.I., and Willsky, A.S. (2011). A sticky HDP-HMM with application to speaker diarization. Ann. Appl. Stat. *5*, 1020–1056.

47. Montanez, G.D., Amizadeh, S., and Laptev, N. (2015). Inertial hidden Markov models: modeling change in multivariate time series. In Twenty-Ninth AAAI Conference on Artificial Intelligence.

48. Denker, M., Yegenoglu, A., and Grün, S. (2018). Collaborative HPC-Enabled Workflows on the HBP Collaboratory Using the Elephant Framework. Neuroinformatics *2018*, 19.

49. Williamson, R.C., Cowley, B.R., Litwin-Kumar, A., Doiron, B., Kohn, A., Smith, M.A., and Yu, B.M. (2016). Scaling properties of dimensionality reduction for neural populations and network models. PLoS Comput. Biol. *12*, e1005141.

50. Semedo, J.D., Zandvakili, A., Machens, C.K., Yu, B.M., and Kohn, A. (2019). Cortical areas interact through a communication subspace. Neuron *102*, 249–259.e4.

51. Pohle, J., Langrock, R., van Beest, F.M., and Schmidt, N.M. (2017). Selecting the number of states in hidden markov models: pragmatic solutions illustrated using animal movement. JABES *22*, 270–293.

52. Kobak, D., Brendel, W., Constantinidis, C., Feierstein, C.E., Kepecs, A., Mainen, Z.F., Qi, X.L., Romo, R., Uchida, N., and Machens, C.K. (2016). Demixed principal component analysis of neural population data. Elife *5*, e10989.

53. Kuhn, H.W., and Yaw, B. (1955). The Hungarian method for the assignment problem. Nav. Res. Logist. Q. *2*, 83–97.

54. Kluyver, T., Ragan-Kelley, B., Pérez, F., Bussonnier, M., Frederic, J., Hamrick, J., Grout, J., Corlay, S., Ivanov, P., Abdalla, S., et al. (2016). Jupyter notebooks—a publishing format for reproducible

CellPress
OPEN ACCESS

computational workflows. In 20th International Conference on Electronic Publishing.

55. Hunter, J.D. (2007). Matplotlib: a 2D graphics environment. Comput. Sci. Eng. *9*, 90–95.

56. Harris, C.R., Millman, K.J., van der Walt, S.J., Gommers, R., Virtanen, P., Cournapeau, D., Wieser, E., Taylor, J., Berg, S., Smith, N.J., et al. (2020). Array programming with NumPy. Nature *585*, 357–362.

57. Reback, J., McKinney, W., Jbrockmendel, X., Bossche, J.V.D., Augspurger, T., Cloud, P., Gfyoung, X., Hawkins, S., Sinhrks, X., Roeschke, M., et al. (2021). pandas-dev/pandas: Pandas 1.2.3 (Zenodo). https://doi.org/10.5281/zenodo.3509134

58. McKinney, W. (2010). Data structures for statistical computing in Python. In Proceedings of the 9th Python in Science Conference (SciPy 2010), pp. 56–61.

59. Hoyer, S., and Hamman, J.J. (2017). xarray: N-D labeled Arrays and Datasets in Python. J. Open Res. Softw. *5*, 10.

60. Waskom, M. (2021) Seaborn: statistical data visualization (Zenodo). https://doi.org/10.21105/joss.03021

61. SciPy 1.0 Contributors, Virtanen, P., Gommers, R., Oliphant, T.E., Haberland, M., Reddy, T., Cournapeau, D., Burovski, E., Peterson, P., Weckesser, W., et al. (2020). SciPy 1.0: fundamental algorithms for scientific computing in Python. Nat. Methods *17*, 261–272.

62. Pedregosa, F., Varoquaux, G., Gramfort, A., Michel, V., Thirion, B., Grisel, O., Blondel, M., Prettenhofer, P., Weiss, R., Dubourg, V., et al. (2011). Scikit-learn: machine learning in Python. J. Mach. Learn. Res. *12*, 2825–2830.

STAR★METHODS

KEY RESOURCES TABLE

REAGENT or RESOURCE	SOURCE	IDENTIFIER
Experimental models: Organisms/strains		
Long-Evans Male Rats	Janvier	N/A
Software and algorithms		
Custom Analysis Code	Bence Bagi	https://doi.org/10.5281/zenodo.5839003
PonyORM	N/A	https://github.com/ponyorm/pony/
ssm	N/A	https://github.com/lindermanlab/ssm
Elephant	N/A	http://neuralensemble.org/elephant/
xarray	N/A	https://xarray.pydata.org/
scikit-learn	N/A	https://scikit-learn.org/
MoviePy	N/A	https://zulko.github.io/moviepy/
Datasets		
Hide-and-seek dataset	Custom	https://doi.org/10.12751/g-node.ib9tty

RESOURCE AVAILABILITY

Lead contact

Further information and requests for resources should be directed to and will be fulfilled by the lead contact, Juan Ignacio Sanguinetti-Scheck (jsanguinettischeck@fas.harvard.edu).

Materials availability

This study did not generate new unique reagents.

Data and code availability

Data have been deposited at a G-Node repository and are publicly available as of the date of publication under the link https://gin.g-node.org/bagibence/hidenseek_hmm. DOIs are listed in the key resources table
Code necessary for reproducing the analysis has been deposited in Zenodo and are publicly available as of the date of publication at https://github.com/bagibence/hidenseek_hmm. DOIs are listed in the key resources table.

EXPERIMENTAL MODEL AND SUBJECT DETAILS

The present study includes data from two studies that introduced hide-and-seek as a model for complex play behavior with male rats (n=8 total). For full description of the training, behavioural methodology, classification of behaviour and protocols (G 0297/18) please see Reinhold et al.[23] and Concha-Miranda et al.[24]

METHOD DETAILS

Video recordings

We acquired wide angle video of the rats' behavior via one or two overhead Flir Chameleon 3 cameras (FLIR® Systems, Inc., USA) running at 30 frames per second. A second Flir Chameleon 3 camera recorded either the room itself or the observer behavior inside the observer cage. The images obtained and the corresponding camera metadata related to frame identity and digital input pin states were recorded using Bonsai[42] performing online tracking of the rat.

Behaviors pertaining to hide-and-seek were manually classified according to Reinhold et al.[23] and Concha-Miranda et al.[24] using the software ELAN.

In the case of the Concha-Miranda et al.[24] paper, when the recorded rat is in the observer position, the experimenters behaviorally tagged the hide-and-seek behaviors of the playing (not recorded) rat, as well the behavior of the observing (recorded) rat into 3 categories (engaged, resting, grooming).

CellPress
OPEN ACCESS

Neural acquisition

We implanted 8 male Long–Evans rats with Harlan-8 tetrode Drives (Neuralynx Inc., USA) in the medial prefrontal cortex (Cg1, PL, IL) at coordinates (Bregma + 3, lateral + 0.5). Tetrodes were arranged in a 2 by 4 matrix resulting in recordings between 2.3 mm and 3.8 mm anterior from bregma and 0.25 mm and 0.75 mm lateral from bregma. Tetrodes were turned from 12.5 μm diameter nichrome wire (California Fine Wire Company) and gold plated to 250–300 kΩ impedance. In order to identify tetrodes in the anatomy of PFC, tetrodes were stained with fluorescent tracers DiI and DiD (ThermoFisher Scientific Inc., USA) before implantation. Before surgery, animals were initially anesthetized with isoflurane (cp-pharma, 31303 Burgdorf) followed by an intraperitoneal injection of ketamine (100 mg/kg, Medistar Arzeneimittelvertrieb, 59387 Aschberg) and xylazine (7.5 mg/kg, WDT, 30827 Garbse). During surgery body temperature was kept at 36°C with a thermal blanket, and a non-traumatic head holder was used. Surgeries took 2–3 hr, after which the animals woke up and were treated with carpofen (5 mg/kg, Zoetis Deutschland GmbH 10785 Berlin) for 2 days. Animals were checked on regularly to ensure proper recovery from the surgery. No complications occurred. After 2–3 days of surgery, rats were gradually re-habituated to play hide-and-seek. The first 2 days, rats freely explored the room or remained as observers while a demonstrator rat played. Once animals showed interest in playing again, the hide-and-seek protocol was restarted.

We recorded neural signals using a 32-channel wire-free neural logger developed by Deuteron Technologies, recording extracellular signals at 32 kHz. The system consists of a headstage performing amplification and digitalization, where the multiplexed signal is then processed in a processor board and stored on a micro SD card on the head of the animal. The whole system is mechanically attached to the cap of the Harlan-8 drive and covered by a protective case that also served as a red target in our online movement tracking.

The processor board of the Neural Logger receives and transmits radio signals allowing for communication with a base station. Radio communication is fast enough to enable synchronization via TTL's between the base station and the logger. Hardware copies of these TTL's are also sent to the cameras via I/O pins.

Extracellular recordings were spike detected and sorted using Kilosort.[43] After the initial sorting, clusters were manually curated using Phy in Python. Cluster quality was assessed by spike shape, cluster separation of its principal components. SNR and ISI-histogram with lack of contamination in a 1 ms refractory period.

After conclusion of the experiment, rats were anesthetized with urethane and depth positions on selected tetrodes were marked with electrolytic lesion. The lesions were conducted using a NanoZ (Neuralynx Inc., USA) with a DC current of −8 (μA) for 8 s, tip negative. The rats then received an overdose of the anaesthetic and were transcardially perfused using a prefix solution followed by PFA at 4%. The brains were extracted and post-fixed in 4% PFA for 18–24 hr before being sectioned coronally into 100 μm thick sections. Before proceeding with tissue staining, slices were photographed at an epi-fluorescence microscope to reveal differential patterns of fluorescence dyes (DiI or DiO) on different tetrodes. This allowed for further identification of each individual tetrode. To finalize the histology tissues went through cytochrome oxidase staining and were imaged under a bright field microscope to visualize the lesions and identify the anatomical location of each tetrode in the brain, in according to the Paxinos & Watson rat brain atlas (Sixth edition, 2007).

QUANTIFICATION AND STATISTICAL ANALYSIS

Selecting sessions for analysis

One session where a single neuron was recorded (session #6) was excluded from the analysis. The analyzed sessions had the following number of recorded neurons: 9, 12, 7, 5, 11, 14, 15, 16, 9, 8, 31, 31, 8. The observing data set had 12 sessions with the following number of recorded neurons: 11, 12, 12, 11, 13, 10, 16, 11, 8, 8, 17, 13.

Segmenting neural data in time

In all our analyses, spikes on each trial are binned into 250 ms long non-overlapping bins.

For Figure 1D spikes are smoothed with a Gaussian window with 500 ms half-width and the resulting firing rates are scaled between 0 and 1 per neuron. K-means clustering is performed with K=6. The number of clusters in time is determined by cross-validation using TimeSeriesSplit in scikit-learn with 20 segments and the kneedle[44] algorithm for finding the knee of the test-score curve. For visualization, neurons are ordered according to a hierarchical clustering, using correlation through time as a distance measure.

The main component of our analysis is segmenting the neural data recorded during the hide-and-seek games in an unsupervised manner by inferring sequences of discrete states underlying the spiking activity in each trial. To fit the HMMs to our data, we used the *ssm* package.[45] For each session, we initialize with 40 different random seeds and pick the model which maximizes the log-likelihood of the observed data. We used "sticky" transitions, increasing the probability of self-transitions, which has been shown to reduce rapid switching of states in a variety of task,[46,47] with the default parameters in *ssm* ($\alpha = 1$, $\kappa = 100$). The effect of "sticky" transitions is similar to what Rubin et al.[22] achieve by filtering out short deviations from clusters.

Additionally, we have found that pre-processing spike trains using GPFA (implemented in the *elephant* Python package[48]) can effectively "denoise" the data and further reduce rapid switching. Therefore, we bin spike times into 250 ms long non-overlapping time bins, and these spike counts are fed to the GPFA algorithm. Next, we fit HMMs on the factors found by GPFA. Since the factors, x_i, are Gaussian processes, HMM observations, y_i, at each time point are normally distributed.

Since the GPFA factors are designed to capture the shared activity in the recorded neuronal population, we attempted to set the number of factors to the dimensionality of the population activity for each recording separately. To determine this dimensionality (i.e.

pick the number of factors of a session), we used the two-step technique based on factor analysis and cross-validation, described in Williamson et al.[49] and also applied by Semedo et al. [50]: (1) We fitted FA models with increasing number of dimensions and picked the model that maximized the cross-validated log-likelihood on the session's firing rates. (2) We picked the GPFA dimensionality as the smallest dimensionality that captured 95% of the variance of the shared covariance matrix, C^TC, of the model from the previous step.

It is generally hard to pick the number of states (K) in hidden Markov models if one does not know it in advance.[51] This is precisely the case in our situation, we have no prior information about the number of activity patterns present in PFC activity. To infer the number of states, we performed cross-validation (10-fold) where we fitted HMMs varying the number of states on a subset of trials in a session, then recorded the log-likelihood of the activity on held-out trials normalized by the number of time points in those trials. Please note that given the behavioral diversity in our experiments from trial-to-trial, we do not think cross-validation can uncover the "ground truth" regarding the number of states. However, testing the values $K \in \{5, \ldots, 45\}$, based on this cross-validation procedure we concluded $K \in \{9, \ldots, 16\}$ to be an appropriate range for the number of HMM states in the reference session, with K=10 maximizing the test-likelihood. Fitting on all trials of the session, we have qualitatively found that adding an additional state (K=11), while being as simple as possible, results in a clearer representation of transit and interaction with the experimenter. Additionally, in our experience models with $K \in \{9, \ldots, 16\}$ produce qualitatively similar results.

Although we should not expect the activity in different sessions to be described by the same number of states, we imposed this number on every session to be able to match states between them.

We emphasize that the methods used here are not critical details of the general approach, we obtain qualitatively similar results fitting HMMs with Poisson observations on the spike counts directly (skipping preprocessing using GPFA), using different bin lengths, or by further embedding the factors using UMAP and applying K-means clustering.

Visualization using UMAP

While UMAP could in principle be applied as a non-linear dimensionality reduction step in the analysis pipeline, here we use it for visualization purposes only, embedding GPFA factors into 3 dimensions with the settings *n_components*=3, *n_neighbors*=40.

Stretching

Trials are stretched to the same length and aligned to each other by first establishing a shared set of reference time points by taking the median across trials for each of the time points that signal a change in the phases of the game, then stretching time in individual trials to align with these using interpolation, also used by Kobak et al.[52] In the case of firing rates linear interpolation is used, while for aligning states (which have discrete values) the value at the nearest time coordinate is taken.

Mutual information

Just as hidden Markov models segment the trials into intervals based on the neural data, the time points tagged based on the videos signaling behavioral changes define another segmentation of the same trials. Treating the two segmentations as two clusterings of the same set of time points, we can use the mutual information (MI) score – a measure of similarity between two clusterings – to quantify their correspondence. In our analysis, we concatenate states – behavioral and HMM separately – from all trials of a session and obtain a single MI score per session.

To test if the segmentation by the HMMs is more similar to the behavioral segmentation than what we could expect by chance, we generate 10.000 "fake segmentations" and a distribution of mutual information scores we could expect if we segmented the trials randomly. These fake clusterings are generated by, for every trial, randomly shuffling the order of the HMM state segments. Finally, segmentations on all trials of a session are concatenated and the MI with the behavioral segmentation is calculated the same way as for the "real" HMM segmentation.

The same random segmentations are used to test the significance of the conditional probabilities in Figure 3D. We calculate, for each combination of HMM- and behavioral states, the conditional probabilities P(HMM state | behavioral state) and P(behavioral state | HMM state). Doing the same using the randomly generated clusterings produces a null-distribution of conditional probabilities we can compare the original values to. When calculating which conditional probabilities are significant, we only consider cases where the real conditional probability is larger than 0.001. To generate Figure S3, we repeated this same analysis for every session, and for each behavioral state counted in how many sessions we found an HMM state for which P(HMM state | behavioral state) was significantly higher than chance.

We further tested for the significance of timing alone by comparing mutual information with cyclic rotations of the HMM segmentation obtained, keeping both the statistics of segment lengths, as well as the statistics of the transitions between them. This was done by concatenating the HMM states from all trials of a session and generating a null-distribution of scores by shifting the state labels by every possible number of time bins.

These bootstrapping analyses are done on each session separately and are independent of the matching procedure.

Another but similar approach is taken when analysing the second, "observing" data set (Figure S6). In this case we use mutual information to quantify the relationship of the inferred states in a trial with the observing or playing role of the animal in that trial. This is done by calculating the mutual information of the HMM states with a binary labeling in which every time point in observing trials is class A, every time point in playing trials is class B. To compare the achieved score to the distribution of MI scores we would get by chance, we keep the transition points of the segmentation inferred by the HMM but randomly relabel every segment (i.e., assign it a class label from {1,..., K}), and calculate the MI score of these random labels with the binary labeling mentioned above.

Decoding analysis

To decode the tagged behavioral states from neural activity, we used a random forest classifier with the default settings in scikit-learn. For testing whether the random forest's performance was above chance level, we compared it to two "dummy classifiers". The dummy classifier with the "stratified" strategy chooses a random label for each data point in the test set by drawing from the distribution of the labels in the training data. The dummy classifier with the "most frequent" strategy predicts the most frequent label in the training data. We performed 10-fold cross-validation, training on 9/10 of the trials and testing on the remaining ones, then compared the distributions of decoding scores using a one-sided Wilcoxon signed-rank test. We performed the test against both the "stratified" and the "most frequent" dummy classifiers and took the larger p-value in each session.

Matching

Because we record a different subset of neurons in different sessions, we have to fit a different HMM to each session. In order to generate Figures 3B and 3C and demonstrate that states that are locked to the phases of the game are found in multiple sessions, we attempted to match states identified on different sessions to each other. To do this, first we calculated for each state the probability of it being active at every time point in each session. We calculated the median values of the time points signaling the phases of the game across every trial of every session – for hide and seek separately in order to include the "box open" point in seek trials. We stretched every trial to the same length by aligning them to these median points. Then to match the states of two sessions, we calculated the correlations of these time series for every pair of states in the two sessions' models, again in hide and seek separately. This results in 2 correlation matrices. We then applied the Hungarian algorithm[53] to the (negative) average of these correlation matrices to find the pairing with the highest total correlations. To pick a reference session, we sum up the correlations between the identified state pairs between every pair of sessions. The session with the highest average correlation to all the other sessions (session #12) is chosen as the reference session. We then permute the states' identities in all the other sessions so that they match the order of the reference session. (e.g., if state #9 of session #5 is matched to the state #1 of the reference session, we relabel the states of session #5 such that what used to be state #9 becomes state #1.)

We tried alternative ways of matching the states such as using other distance measures between the time series, as well as using contingency matrices between behavioral and HMM states instead of state probabilities in time, with similar results as this method.

Video summary figures

In Figure 4 we aim to summarize what happened in the videos while particular neural states were active. In short, we extract the parts of the videos where the state was active and combine the frames into a single image by stacking them on top of each other. More precisely, first we extract a frame on which neither the animal, nor the experimenter are present and use it as a background. Then for Figures 4B–4D, for – to avoid overlapping extractions – every second frame when the neural state was active, we identify the animal's location and extract a bounding box around it from the video frame. Finally, we overwrite the background image at this location with the part extracted from the frames.

For Figures 4A and S5 a slightly different technique is used. Here, instead of based on the animal's position, we try to identify positions in the frames where something "interesting" happened. To do this, for every second frame when the state is active, we calculate the structural similarity score between the background and the frame at each pixel, use adaptive thresholding to binarize the image, then find patches where they are different. We tag these areas as "interesting" and used them to overwrite the background image's corresponding pixels. This way, areas where the frame is different from the background are included in the final summary image. This way not only the rat's but also the experimenter's activity can be potentially included in the final summary image.

Software tools

All analyses were done in the Python programming language, making extensive use of the following libraries: *Jupyter Notebooks*,[54] *matplotlib*,[55] *numpy*,[56] *pandas*,[57,58] *xarray*,[59] *seaborn*,[60] *scipy*,[61] *scikit-learn*,[62] *PonyORM*, *ssm*,[45] *elephant, MoviePy*.

Current Biology, Volume *32*

Supplemental Information

Unsupervised discovery of behaviorally relevant brain states in rats playing hide-and-seek

Bence Bagi, Michael Brecht, and Juan Ignacio Sanguinetti-Scheck

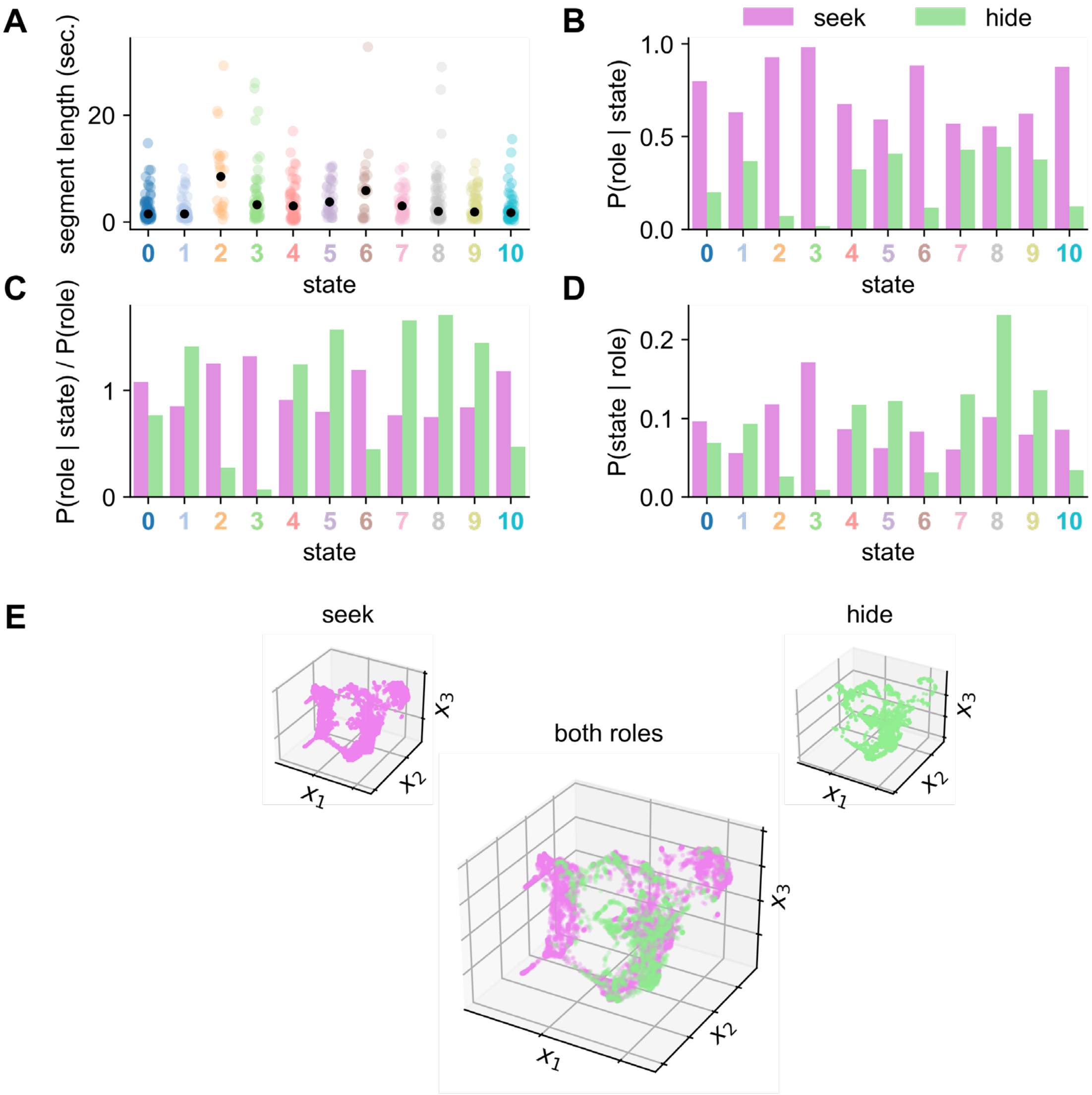

Figure S1) State segment lengths and state usage in the hide-and-seek roles in the reference session. **Related to Figure 2. A)** Segment lengths for each HMM state. Median values indicated in black. **B)** P(role | state) **C)** P(role | state) / P(role). This normalization takes into account that seek trials are longer and hence more probable. **D)** P(state | role) **E)** UMAP embeddings of neural activity colored by hide-and-seek role.

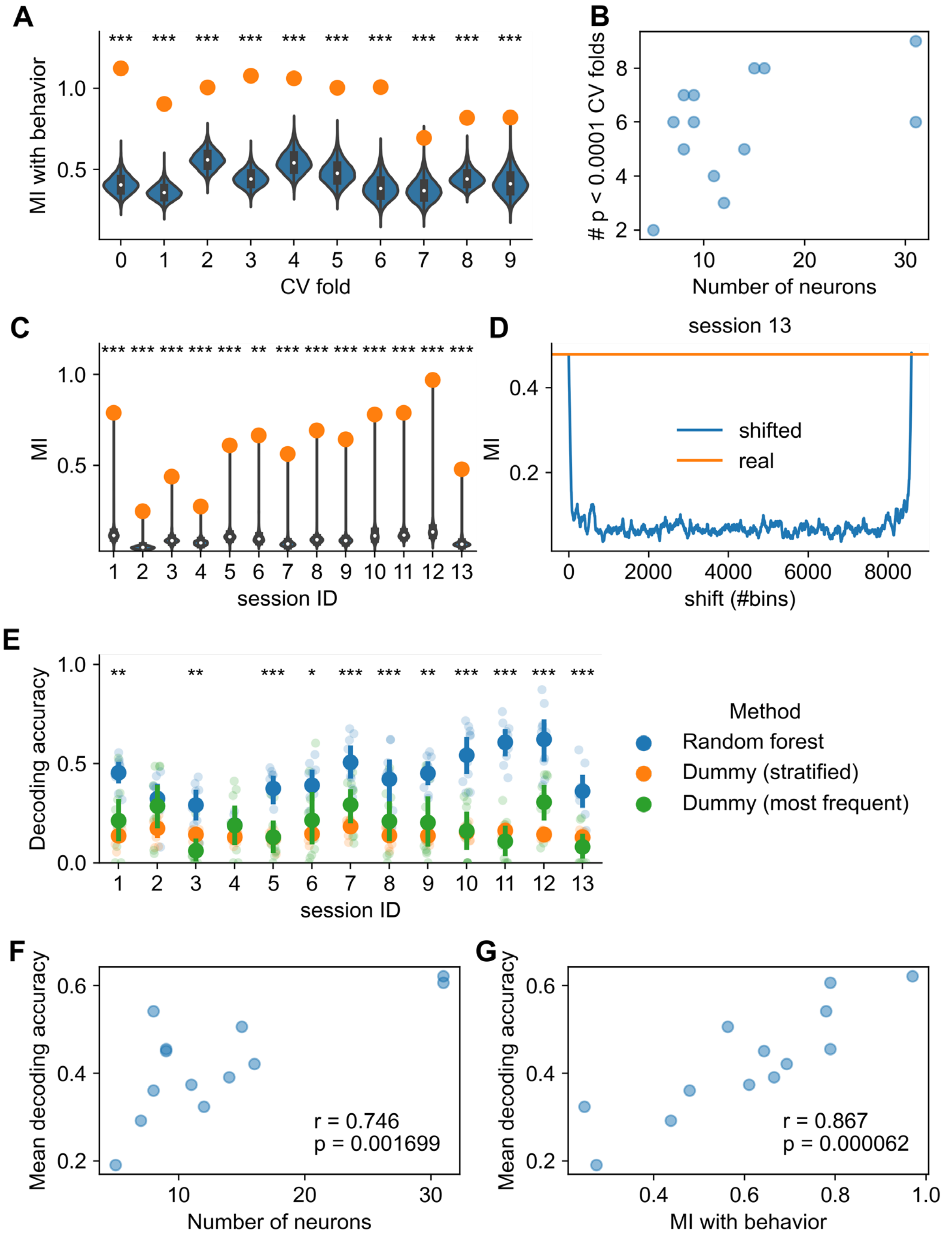

Figure S2) Cross-validated mutual information analysis and supervised decoding of behavioral states. Related to Figure 2. A) Fitting an HMM on a subset of trials, then applying on held-out

trials and performing the same shuffling analysis as in Fig 2E on the reference session. The star-code shows the p-value for the bootstrap with *=0.05, **=0.01, ***=0.001 **B)** Number of significant cross-validation folds (out of 10 folds) per session as a function of the number of recorded neurons in that session (using $p < 0.0001$, as this level was found to be true for every session without cross-validation in Fig 2E). **C)** Mutual information of HMM states vs behavior analysis (orange dot) for all sessions compared to a circular shuffling of the HMM states by all possible time-shifts (blue distribution) **D)** Figure shows for session #12 how the consecutive shifts affect mutual information. **E)** Cross-validated decoding of behavioral states from GPFA factors (10-fold CV across trials) using a random-forest classifier performs better than chance (performance of dummy classifiers) (one-sided Wilcoxon signed-rank test). The star-code shows the p-value with *=0.05, **=0.01, ***=0.001 **F)** Mean decoding accuracy increases with the number of neurons. (Pearson correlation, one-sided test). **G)** Mean decoding accuracy increases with the MI with behavior on the same session. (Pearson correlation, one-sided test.)

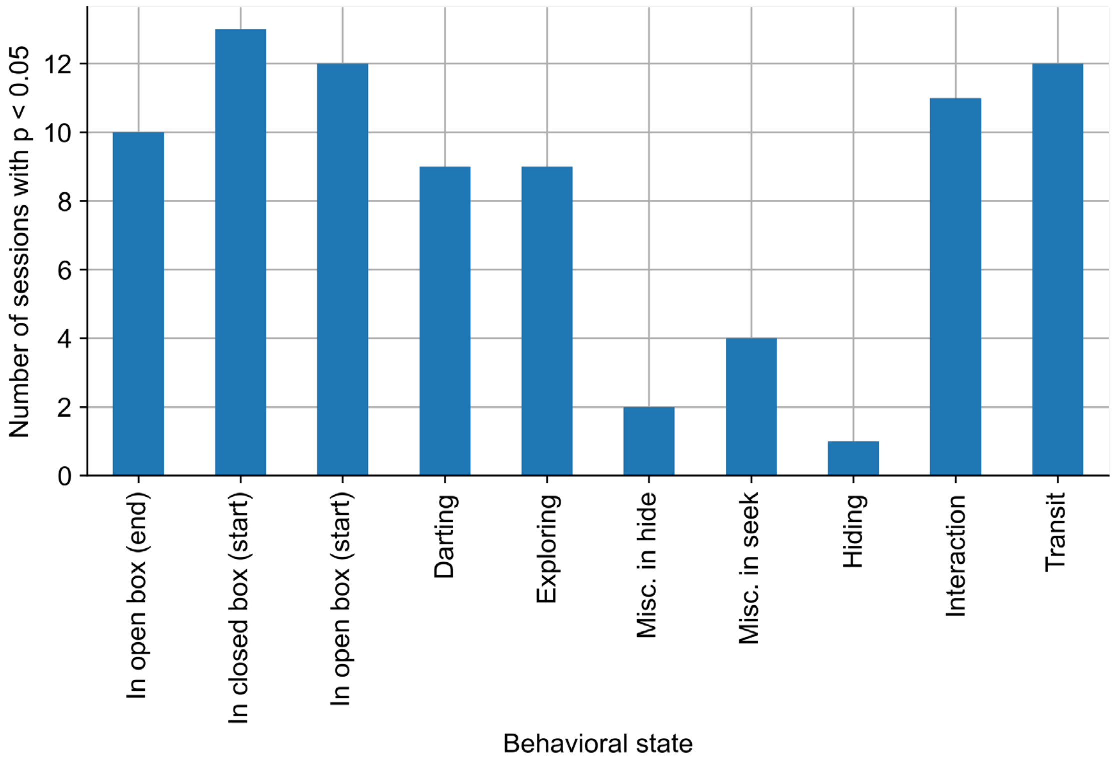

Figure S3) Significant sessions per behavior. Related to Figure 3. The number of sessions (out of 13) in which there is an HMM state significantly correlated with a tagged behavioral state ($p < 0.05$, Holm-Bonferroni correction for multiple comparisons)

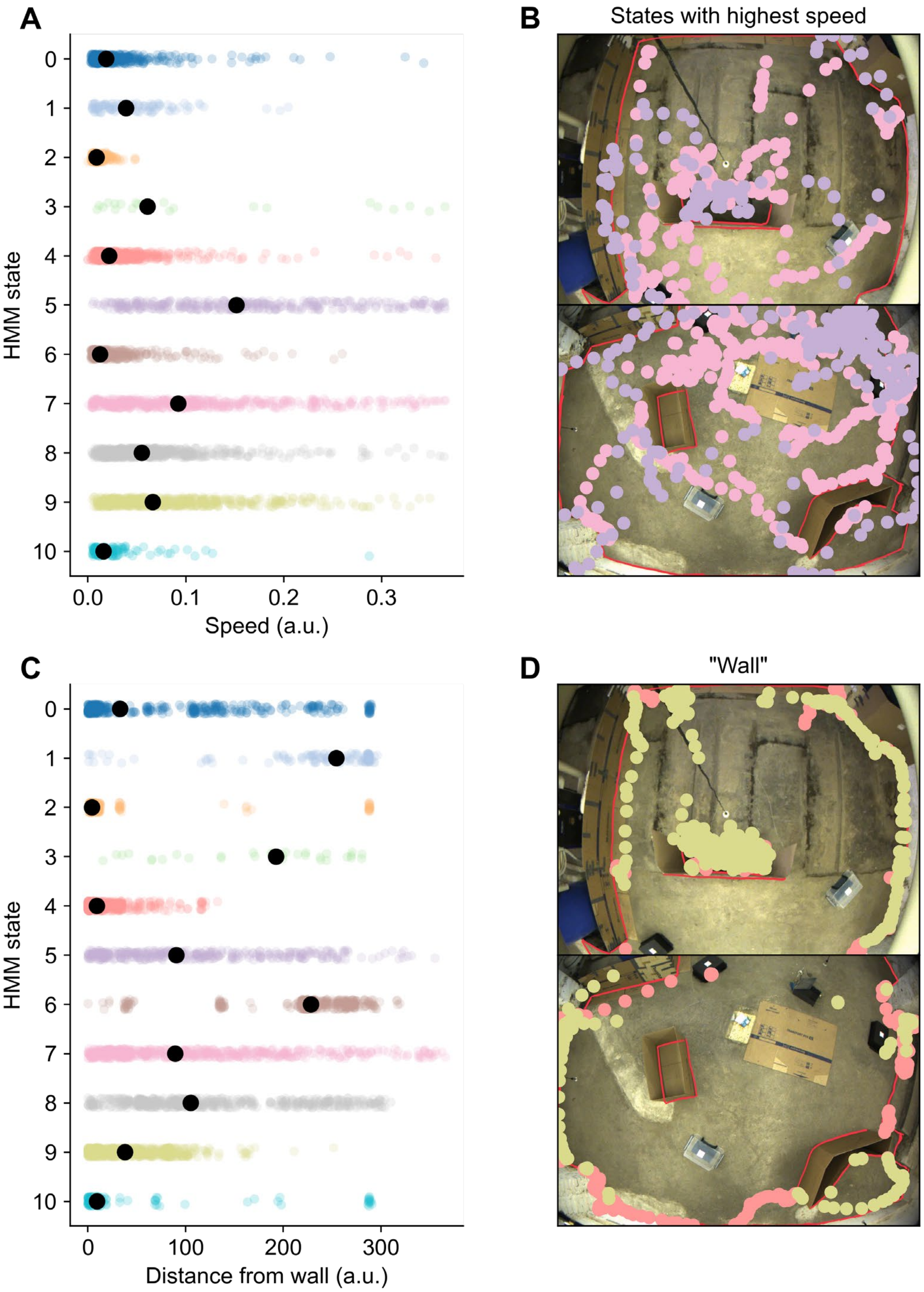
A
B
States with highest speed
HMM state
0
1
2
3
4
5
6
7
8
9
10
0.0
0.1
0.2
0.3
Speed (a.u.)
C
D
"Wall"
HMM state
0
100
200
300
Distance from wall (a.u.)

Figure S4) Quantification of the "wall" and "running" states. Related to Figure 4. A) Animal's approximate speed when each state was active. One data point per timestep. **B)** Animal's position when states #7 (running) and #5 (transit) were active. **C)** Animal's approximate distance from the nearest wall when each state was active. **D)** Animal's positions when states #9 and #4 were active. These states were active when the animal was walking along (#9 and #4) and stopping at (#4) walls.

A

B

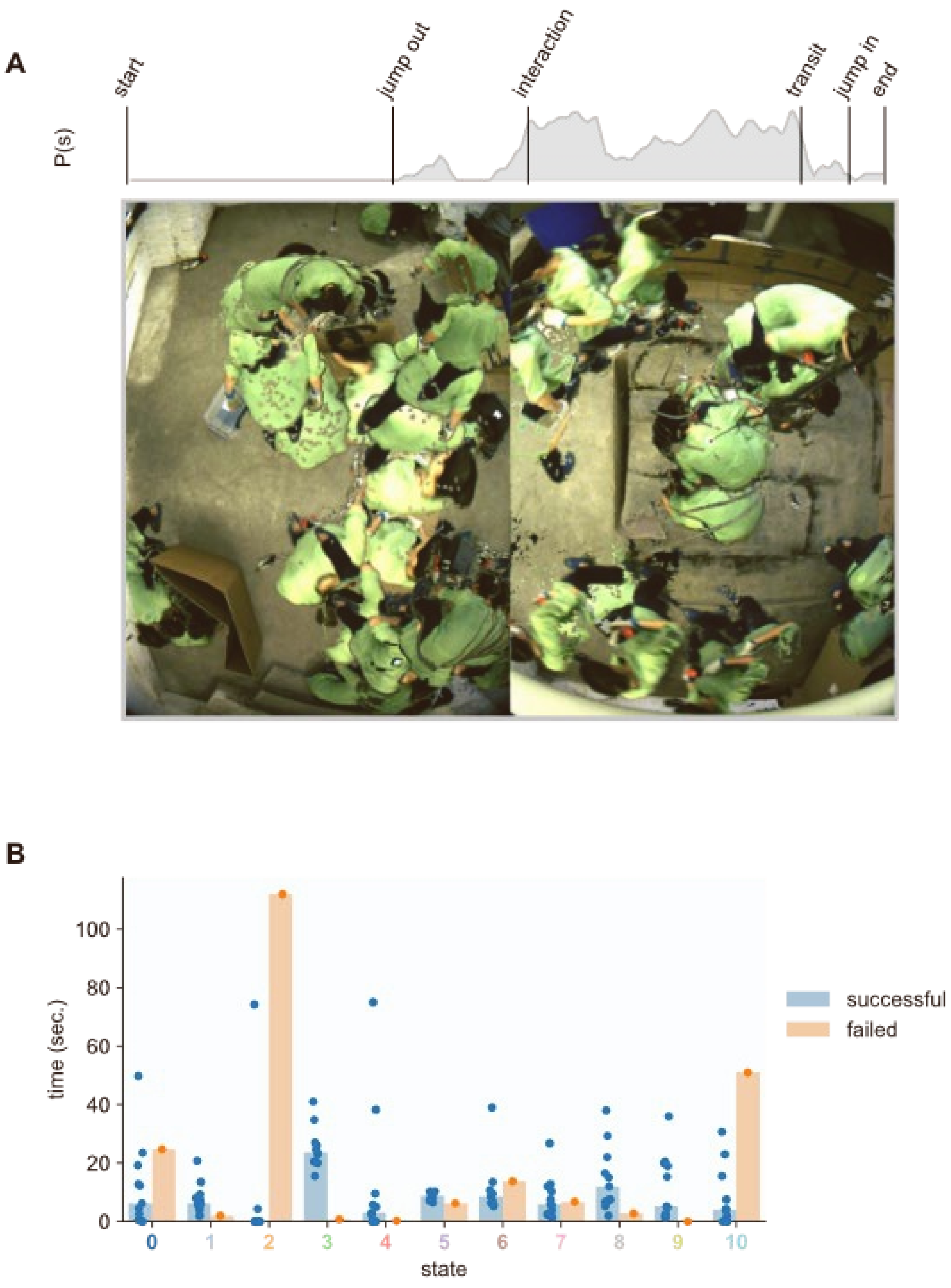
start
jump out
interaction
transit
jump in
end
P(s)
time (sec.)
0
20
40
60
80
100
state
0
1
2
3
4
5
6
7
8
9
10
successful
failed

Figure S5) Interaction-related state and its absence in failed trials. Related to Figure 4. A) Condensed video frames during state #8 show interaction with the experimenter. Marginal: probability of the state #8 throughout the trial, averaged across all trials of the reference session. **B)** Time spent in the different states for the reference session on failed and successful trials of seek behavior. Note that for successful trials state #8 (playing with the experimenter) has significantly longer durations than for the single failed seek trial. Note that the failed trial is dominated by states #2 and #10 that we have post-hoc discovered as resting states.

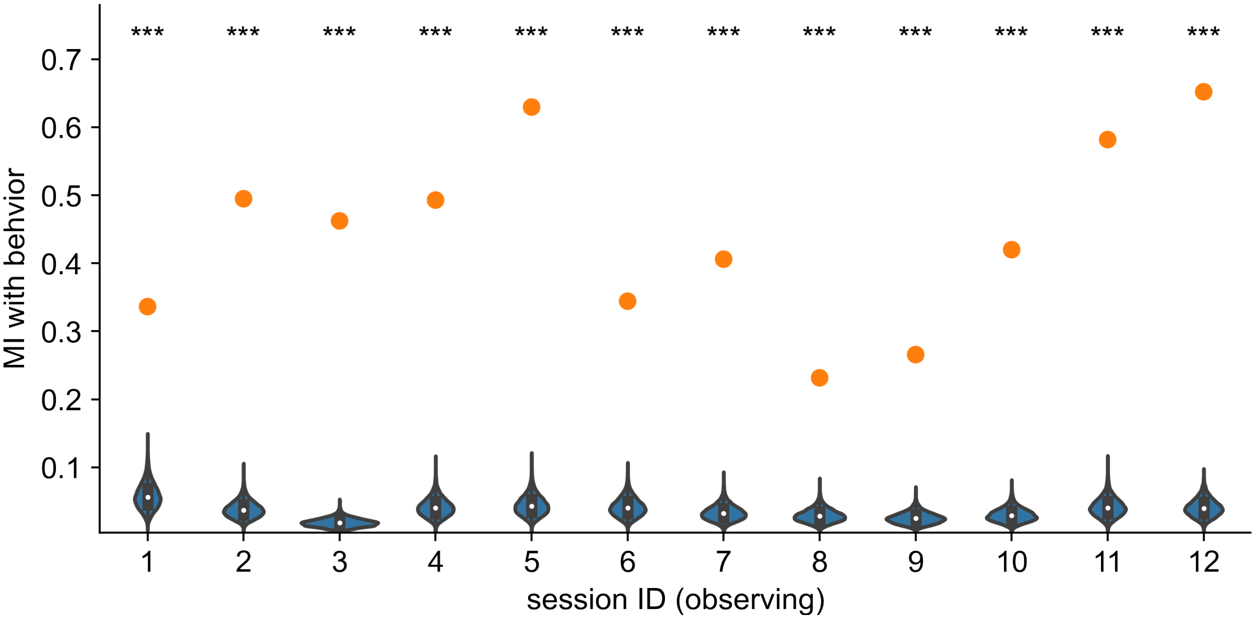

Figure S6) Separation between playing and observing. Related to Figure 6. The separation between playing- and observing states, quantified by mutual information, is well above chance level ($p < 0.0001$ in all sessions). The star-code shows the p-value for the bootstrap with *=0.05, **=0.01, ***=0.001
